# Integrative multi-omics analysis identifies a prognostic miRNA signature and a targetable miR-21-3p/TSC2/mTOR axis in metastatic pheochromocytoma/paraganglioma

**DOI:** 10.7150/thno.35458

**Published:** 2019-07-09

**Authors:** Bruna Calsina, Luis Jaime Castro-Vega, Rafael Torres-Pérez, Lucía Inglada-Pérez, Maria Currás-Freixes, Juan María Roldán-Romero, Veronika Mancikova, Rocío Letón, Laura Remacha, María Santos, Nelly Burnichon, Charlotte Lussey-Lepoutre, Elena Rapizzi, Osvaldo Graña, Cristina Álvarez-Escolá, Aguirre A de Cubas, Javier Lanillos, Alfonso Cordero-Barreal, Ángel M Martínez-Montes, Alexandre Bellucci, Laurence Amar, Fabio Luiz Fernandes-Rosa, María Calatayud, Javier Aller, Cristina Lamas, Júlia Sastre-Marcos, Letizia Canu, Esther Korpershoek, Henri J Timmers, Jacques WM Lenders, Felix Beuschlein, Martin Fassnacht-Capeller, Graeme Eisenhofer, Massimo Mannelli, Fátima Al-Shahrour, Judith Favier, Cristina Rodríguez-Antona, Alberto Cascón, Cristina Montero-Conde, Anne-Paule Gimenez-Roqueplo, Mercedes Robledo

**Affiliations:** 1 Hereditary Endocrine Cancer Group, Human Cancer Genetics Program, Spanish National Cancer Research Centre (CNIO), Madrid, Spain; 2 INSERM, UMR970, Paris-Cardiovascular Research Center, Equipe Labellisée par la Ligue contre le Cancer, Paris, France; 3 Université Paris Descartes, PRES Sorbonne Paris Cité, Faculté de Médecine, Paris, France; 4 Centro de Investigación Biomédica en Red de Enfermedades Raras (CIBERER), Madrid, Spain; 5 Assistance Publique Hôpitaux de Paris, Hôpital Européen Georges Pompidou, Service de Génétique, Paris, France; 6 Sorbonne Université, Pitié-Salpêtrière Hospital, Department of nuclear medicine, Paris, France; 7 Department of Experimental and Clinical Biomedical Sciences, University of Florence, Florence, Italy; 8 Bioinformatics Unit, Structural Biology Program, Spanish National Cancer Research Center (CNIO), Madrid, Spain; 9 Servicio de Endocrinología y Nutrición, Hospital Universitario La Paz, Madrid, Spain; 10 Assistance Publique-Hôpitaux de Paris, Hôpital Européen Georges Pompidou, Service de Radiologie, Paris, France; 11 Assistance Publique-Hôpitaux de Paris, Hôpital Européen Georges Pompidou, Hypertension Unit, Paris, France; 12 Department of Endocrinology and Nutrition Service, Hospital Universitario 12 de Octubre, Madrid, Spain; 13 Department of Endocrinology, Puerta de Hierro University Hospital, Madrid, Spain; 14 Department of Endocrinology, Albacete University Hospital Complex, Albacete, Spain; 15 Department of Endocrinology, Virgen de la Salud Hospital-Toledo Hospital Complex, Toledo, Spain; 16 Department of Pathology, Erasmus Medical Center Cancer Institute, University Medical Center Rotterdam, Rotterdam, The Netherlands; 17 Department of Internal Medicine, Radboud University Medical Centre, 6525 HP Nijmegen, The Netherlands; 18 Department of Medicine III, University Hospital and Medical Faculty Carl Gustav Carus, Technische Universität Dresden, Dresden, Germany; 19 Medizinische Klinik und Poliklinik IV, Klinikum der Universität München, Munich, Germany; 20 Klinik für Endokrinologie, Diabetologie und Klinische Ernährung, Universitätsspital Zürich, Zürich, Switzerland; 21 Department of Internal Medicine I, Endocrine and Diabetes Unit, University Hospital Würzburg, University of Würzburg, Germany; 22 Comprehensive Cancer Center Mainfranken, University of Würzburg, Germany; 23 Institute of Clinical Chemistry and Laboratory Medicine, University Hospital Carl Gustav Carus, Medical Faculty Carl Gustav Carus, Technische Universität Dresden, Dresden, Germany

**Keywords:** multi-omic integration, pheochromocytoma/paraganglioma, miR-21-3p, liquid biopsy, prognostic biomarker

## Abstract

**Rationale**: Pheochromocytomas and paragangliomas (PPGLs) are rare neuroendocrine tumors that present variable outcomes. To date, no effective therapies or reliable prognostic markers are available for patients who develop metastatic PPGL (mPPGL). Our aim was to discover robust prognostic markers validated through *in vitro* models, and define specific therapeutic options according to tumor genomic features.

**Methods**: We analyzed three PPGL miRNome datasets (n=443), validated candidate markers and assessed them in serum samples (n=36) to find a metastatic miRNA signature. An integrative study of miRNome, transcriptome and proteome was performed to find miRNA targets, which were further characterized *in vitro*.

**Results**: A signature of six miRNAs (miR-21-3p, miR-183-5p, miR-182-5p, miR-96-5p, miR-551b-3p, and miR-202-5p) was associated with metastatic risk and time to progression. A higher expression of five of these miRNAs was also detected in PPGL patients' liquid biopsies compared with controls. The combined expression of miR-21-3p/miR-183-5p showed the best power to predict metastasis (AUC=0.804, *P*=4.67·10^-18^), and was found associated *in vitro* with pro-metastatic features, such as neuroendocrine-mesenchymal transition phenotype, and increased cell migration rate. A pan-cancer multi-omic integrative study correlated miR-21-3p levels with TSC2 expression, mTOR pathway activation, and a predictive signature for mTOR inhibitor-sensitivity in PPGLs and other cancers. Likewise, we demonstrated *in vitro* a *TSC2* repression and an enhanced rapamycin sensitivity upon miR-21-3p expression.

**Conclusions**: Our findings support the assessment of miR-21-3p/miR-183-5p, in tumors and liquid biopsies, as biomarkers for risk stratification to improve the PPGL patients' management. We propose miR-21-3p to select mPPGL patients who may benefit from mTOR inhibitors.

## Introduction

Pheochromocytomas and paragangliomas (PPGLs) are rare neuroendocrine tumors (3-8 cases per 10^6^ person-year) with marked genetic heterogeneity 1. Approximately 15-20% of patients with PPGLs develop metastases, and the 5-year overall survival rate after diagnosis of the first metastasis is 60% 2, 3. Altough clinical characteristics such as tumor size, extra-adrenal location and increased plasma concentration of 3-methoxytyramine could provide useful information to estimate the likelihood of metastasis 4-6, there are no histological criteria or clinically validated molecular biomarkers to identify patients whose tumors will become metastatic. Currently, *SDHB* mutation status is the only genetic factor associated with poor prognosis 7. We recently reported that assessment of telomerase activation and *ATRX* mutations in tumor tissue may have clinical significance for identifying metastatic disease (mPPGLs) 8. Nevertheless, most mPPGLs are currently diagnosed at advanced stages, thus precluding successful management of the disease 9. Furthermore, no effective therapies are available for patients who develop mPPGL 10.

High-throughput omic platforms are robust technologies for identifying molecular mechanisms of tumorigenesis and potential markers for diagnosis, prognosis and response to treatment. In PPGLs, genomic profiling has led to the identification of several markers related to clinical variables, including mPPGL 11-13.

miRNAs regulate gene expression at the post-transcriptional level by targeting mRNAs for degradation and/or repressing their translation. Altered expression of miRNAs has been shown to be paramount for cancer-related processes, becoming a new class of cancer biomarkers detectable in tumor tissues and circulation. In PPGLs patients, some miRNAs have been associated with genotype or mPPGL 12, 14-21. However, most series studied so far were small with limited follow-up, and lacked validation, which hinders the identification of miRNAs with prognostic value.

Here, we perform a comprehensive analysis of miRNomes from 443 PPGLs with clinical and genetic information, aimed at identifying miRNAs predictive for mPPGL. We provide evidence of a metastatic signature, assessed in tumors and liquid biopsies, with prognostic value. Furthermore, integrative miRNA-mRNA analysis, TCGA proteomics and *in vitro* assays support a new role of miR-21-3p in mTOR pathway regulation, with potential therapeutic implications for mPPGL (Figure S1).

## Methods

### miRNA discovery

*Discovery of differentially expressed miRNAs.* Three different public datasets 12, 16, 17 were used for extracting miRNA expression data from 443 samples and computing miRNA differential expression between metastatic and non-metastatic groups in each sub-series. Full details on bioinformatics analyses and filtering criteria applied for selecting miRNAs for validation are provided in Supplementary Methods.

*Confirmation of differentially expressed miRNAs in a validation series*. Validation of selected miRNAs was performed in 49 genetically characterized formalin-fixed paraffin-embedded primary tumors. Patients provided informed consent for the use of specimens and clinical data in accordance with the guidelines of the institutional ethics committees. Furthermore, a set of eight paired primary-metastatic samples was used to assess miRNAs with significant association in the validation step. The main characteristics of this series are presented in Table S1. RNA extraction and miRNA quantification are detailed in Supplementary Methods. One-sided nonparametric Mann-Whitney test (GraphPad Prism, RRID:SCR_002798) was used to confirm selected miRNAs deregulation in validation series, based on the assumption that miRNAs had to follow the same direction as in the discovery series.

*Clinical outcome analysis of validated miRNAs.* Time to progression (TTP) was evaluated with the Kaplan-Meier method and differences between the groups were tested with the log-rank test (SPSS, RRID:SCR_002865) for each validated miRNA. TTP was defined as the time between the first PPGL diagnosis and the first documented metastases. We included 246 patients from the discovery and validation series, either presenting metachronous metastases (presented ≥ one year after diagnosis), or with more than one-year follow-up (median follow-up time=1290 days). Patients without evidence of metastases were censored at the date of the last follow-up.

*Risk model of metastasis prediction.* The models were constructed using stepwise conditional logistic regression with the total cohort of 492 patients (443 from the discovery set plus 49 from the validation series). miRNAs associated with a shorter TTP were included in the analysis. To combine the data, the expression of each miRNA was expressed as a dichotomous variable using the median miRNA expression as the cutoff in each series. Additionally, we included binary variables that identified the different series and the *SDHB* status. The classification power of the model was evaluated by computing receiver operating characteristic (ROC) curves and area under the ROC curves (AUC).

### Integration of miRNA and mRNA expression data: *Targetome*

*Integration of miRNA and mRNA expression profiles of the discovery series.* To identify genes potentially regulated by miR-183-3p and miR-21-3p, we considered only samples from the discovery series with miRNA and mRNA expression data available (n=434), and assumed a regulatory role for a miRNA only if the expression level of the miRNA and its known mRNA target showed a significant negative correlation (*P*<0.05). Analysis and interpretation were carried out in three steps:

Step 1. Generation of a list of candidate target genes for each miRNA from the validated miRNA-target interactions (1027 for miR-183-5p and 853 for miR-21-3p) extracted from miRTarBase release 6.1 22 (http://mirtarbase.mbc.nctu.edu.tw/), OncomiRDB 23 (http://lifeome.net/database/oncomirdb/) and TarBase v.7.0 24 (http://diana.imis.athena-innovation.gr/DianaTools/index.php?r=tarbase/index).

Step 2. Generation of normalized mRNA expression matrices including only step 1 genes (*Targetome*) for each sub-series. Further details are specified in Supplementary Methods.

Step 3. Identification of potential target mRNAs by matching the expression of the miRNAs and their predicted target genes. Spearman's correlations between the six *Targetome* matrices considering each of the three sub-series and the expression of each of the two miRNAs were calculated using R. We further examined genes with significant correlations (*P*<0.05) and ρ<-0.4 in at least one series. Of these genes, only those with either a significant negative correlation in both other remaining series or represented by at least two probes in one sub-series, were considered. Finally, only genes proposed in the literature as potential tumor suppressors or involved in differentiation were taken into account for further consideration in the validation series and the cell model.

*Expression of potential target genes in the validation series.* Candidate target genes selected in the integration analysis were quantified by qPCR in the 49 tumor samples from the validation series as described in Supplementary Methods. Spearman's correlation analysis of mRNA-miRNA expression was performed.

### Pan-cancer targeted integrative analysis of miRNA, mRNA and functional proteomics

We included 8,960 human cancer samples representing 32 different major tumor types for which TCGA data on miRNA expression was available. RNA-seq, miRNA-seq and reverse-phase protein array (RPPA) data were downloaded from UCSC Xena (http://xena.ucsc.edu/) and cBioPortal (http://www.cbioportal.org/). Spearman's correlation coefficient was used to assess the correlation of miRNA and gene/protein levels (SPSS). Results representations were obtained using the ggplot2 R package (RRID:SCR_014601).

*PI3K/AKT/mTOR drug sensitivity signature.* We applied the sensitivity signature scores for the TCGA dataset reported by Zhang et al. 25, which were derived from the signature obtained in cell lines after treatment with PI3K/AKT/mTOR inhibitors 26, and performed a correlation analysis between miR-21-3p expression and the PI3K/AKT/mTOR drug sensitivity signature.

### Functional assays in cell models

*Plasmids and cell lines.* The MIMIC Inducible Human Lentiviral microRNA vector with the mCMV promoter, the TurboGFP reporter and hsa-miR-183-5p (VSH6904-224645044, Dharmacon), and the MIMIC Inducible Human Lentiviral microRNA vector with the mCMV promoter, the TurboRFP reporter and hsa-miR-21-3p (VSH6904-224638696, Dharmacon), were stably introduced into the previously generated and authentified human neuroblastoma cell lines SK-N-AS wild-type (WT) and SK-N-AS SDHB KD (*SDHB* knocked down) 27. SMARTvector Inducible Non-targeting Controls (VSC6570 and VSC6571) were used as controls. Transduced cells were selected using puromycin at 1 μg/ml, and cells with high expression of the reporter were FACS sorted and used for subsequent studies.

Cells were maintained in complete Dulbecco's Modified Eagle Medium (DMEM; Sigma#D5796), supplemented with 10% (v/v) fetal bovine serum (FBS, Sigma), 1% (v/v) penicillin/streptomycin, 0.6% (v/v) Fungizone (Gibco), puromycin (1 μg/ml) and G418 (0.3 mg/ml), and maintained at 37°C/5% CO_2_. Cells were routinely tested for mycoplasma and all experiments were performed between passage 10 and 20. miRNA and reporter expression was induced by treatment with 1 μg/ml of doxycycline; this concentration was selected following optimization experiments.

*Wound healing migration assay.* Experiments were performed as described in Rapizzi *et al.* 28. Cell migration was quantified using *ImageJ* software (RRID: SCR_003070) at 15h and 24h after scratching, based on at least 18 pictures for each time point and condition. Experiments were performed in duplicate.

*qPCR.* Expression of neuroendocrine-to-mesenchymal transition (neuroendoMT) markers and potential miRNA gene targets was evaluated in the cell model by qPCR (Supplementary Methods).

*Western blotting.* Cells were seeded and treated as for RNA extraction, and total protein was extracted and homogenized in RIPA lysis buffer (Sigma) supplemented with protease and phosphatase inhibitors (Sigma). Protein concentration was measured using the Pierce BCA Protein Assay Kit (Thermo Scientific) and total proteins (20 μg) were separated by 10% SDS-PAGE and transferred onto a polyvinylidene difluoride membrane (Millipore, Darmstadt, Germany). Primary antibodies used in this study were rabbit anti-Tuberin/TSC2 mAb (1:1000; Cell Signaling #4308, RRID: AB_10547134), and mouse anti-β-Actin mAb (1:2000; Sigma#A5441, RRID: AB_476744). Absolute band intensities of the indicated proteins were captured and quantified with the Image Lab software v4.1 (BioRad, RRID: SCR_014210). β-Actin was used as loading control normalizer. Data were expressed in relative units using the control condition as reference.

*Drug treatment.* Doxycycline-pretreated cells (120h) were plated in 96-well plates at a density of 2000 cells/well and treated with different concentrations of rapamycin (Apollo Scientific #BIR8101) 24h later. The treatment duration was twice the cell doubling time (~96h) and the CellTitler-Glo (Promega) cell viability assay was used to assess cell viability by luminescence measurement using a Victor plate reader (PerkinElmer) 30 min after addition of the reagent.

### Detection of circulating miRNAs

Serum samples from an independent exploratory series of 36 PPGL patients were enrolled by the COMETE network (France). Authorization was obtained from the institutional review board [Comité de Protection des Personnes, Ile de France III, June 2012]. Informed consent for studies on circulating miRNAs was signed by all patients. Clinical data from this series and the control cohort of adult healthy volunteers (n=10) is summarized in Table S2.

For the preparation of conditioned media (CM), wild type and *Sdhb*-/- immortalized mouse chromaffin cells (imCCs) 29 were seeded under standard culture conditions (DMEM/Glutamax, 10% FBS and 1% penicillin-streptomycin) and maintained at 5% CO2/37°C. Once cells reached an optimal confluence, the monolayer was washed with serum-free (SF) DMEM, and cells were replenished with SF media. After 24h, CM was collected, centrifuged and cells were counted.

For miRNA extraction with mirVana™ PARIS™ Kit (Thermo Fisher Scientific, Waltham, MA, USA), 450 µl of serum or filtered CM (0.2 µm pore size) from cultured cells were used. Droplet digital PCR was utilized for detection and quantification of circulating miRNAs as explained in Supplementary Methods. One-sided nonparametric Mann-Whitney test (GraphPad Prism) was used to test for differences in miRNAs levels in circulation between the different groups.

## Results

### miRNA profiling uncovers a signature of six miRNAs associated with metastatic risk

**Figure 1:**
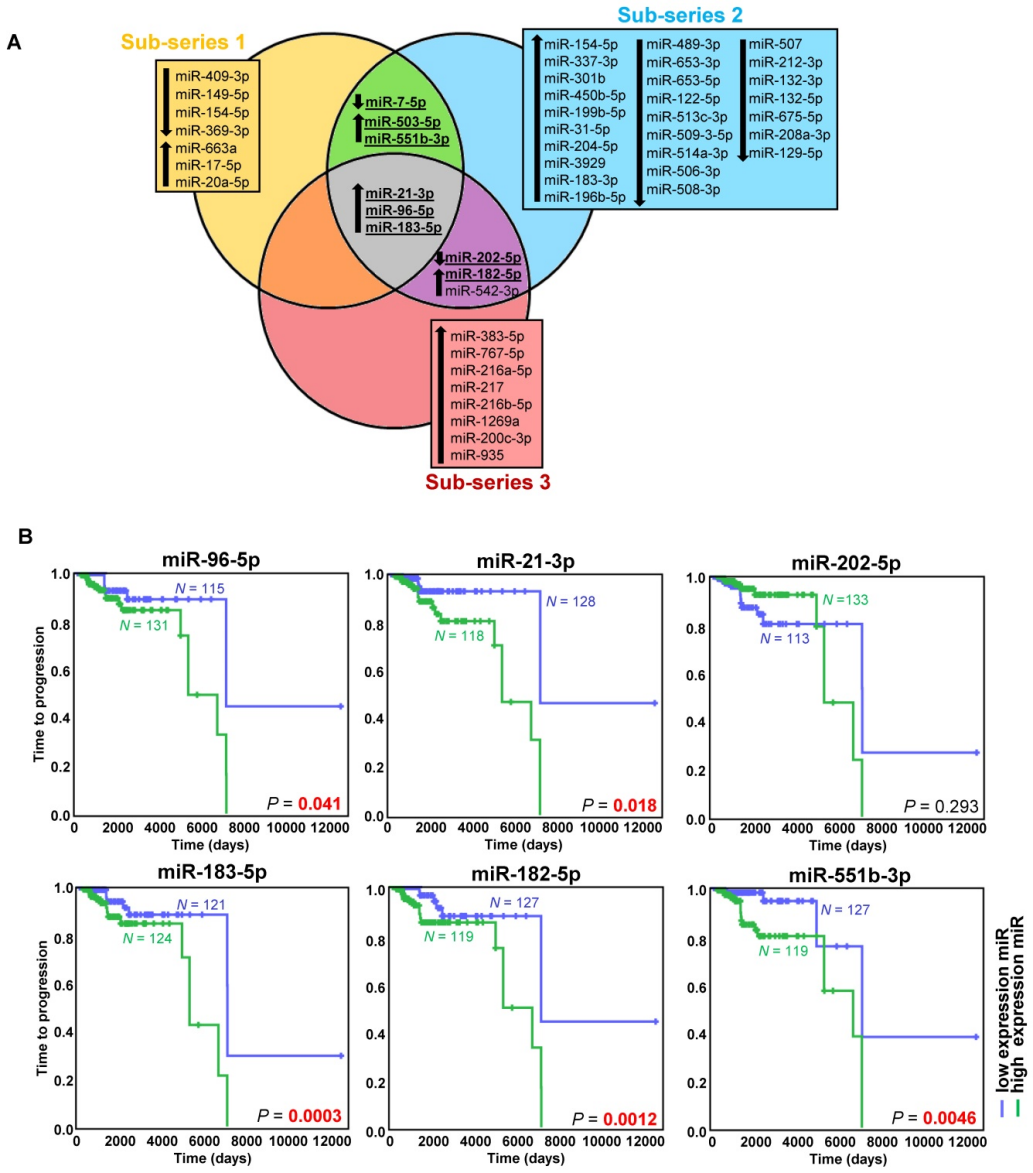
** miRNAs associated with metastatic behavior. (A)** Venn diagram of differentially expressed miRNAs (FDR<0.05, |log_2_ fold change| ≥ 0.75) in the different sub-series of the discovery series (*n*=443). Downregulated miRNAs are indicated with a downward pointing arrow, and upregulated miRNAs are indicated with an upward pointing arrow. miRNAs selected for validation are shown in bold and are underlined. **(B)** Kaplan-Meier plots of time to progression (time between the first PPGL diagnosis and the first documented metastases) of patients according to the expression level of the indicated miRNA in tumor tissue. High expression (above the median expression level of the whole group) of the miRNA is represented in blue and low expression (below the median level) in green (n=246 patients from the discovery and validation series, either presenting metachronous metastases ─ presented ≥ one year after diagnosis ─, or with more than one-year follow-up - median follow-up time=1290 days). Patients without evidence of metastases were censored at the date of the last follow-up. *P*-values were calculated with a log-rank test. *N*: number of patients.

Exploring the discovery series, we identified 49 miRNAs differentially expressed in metastatic compared to non-metastatic cases (Figure 1A, Figure S2A, Table S3). After an extensive literature review (Table S4), six out of the eight selected miRNAs were validated (miR-21-3p, miR-182-5p, miR-96-5p, miR-551b-3p, miR-183-5p, miR-202-5p) (Figure S2B, Table S3).

High expression of the five upregulated miRNAs was significantly associated with a shorter TTP (Figure 1B). Using the paired primary-metastatic tissues, we observed that these five miRNAs showed similar levels in both tissues, and notably miR-21-3p was significantly upregulated in metastases versus primary tumors (*P*=0.0099) (Figure S2B).

**Figure 2:**
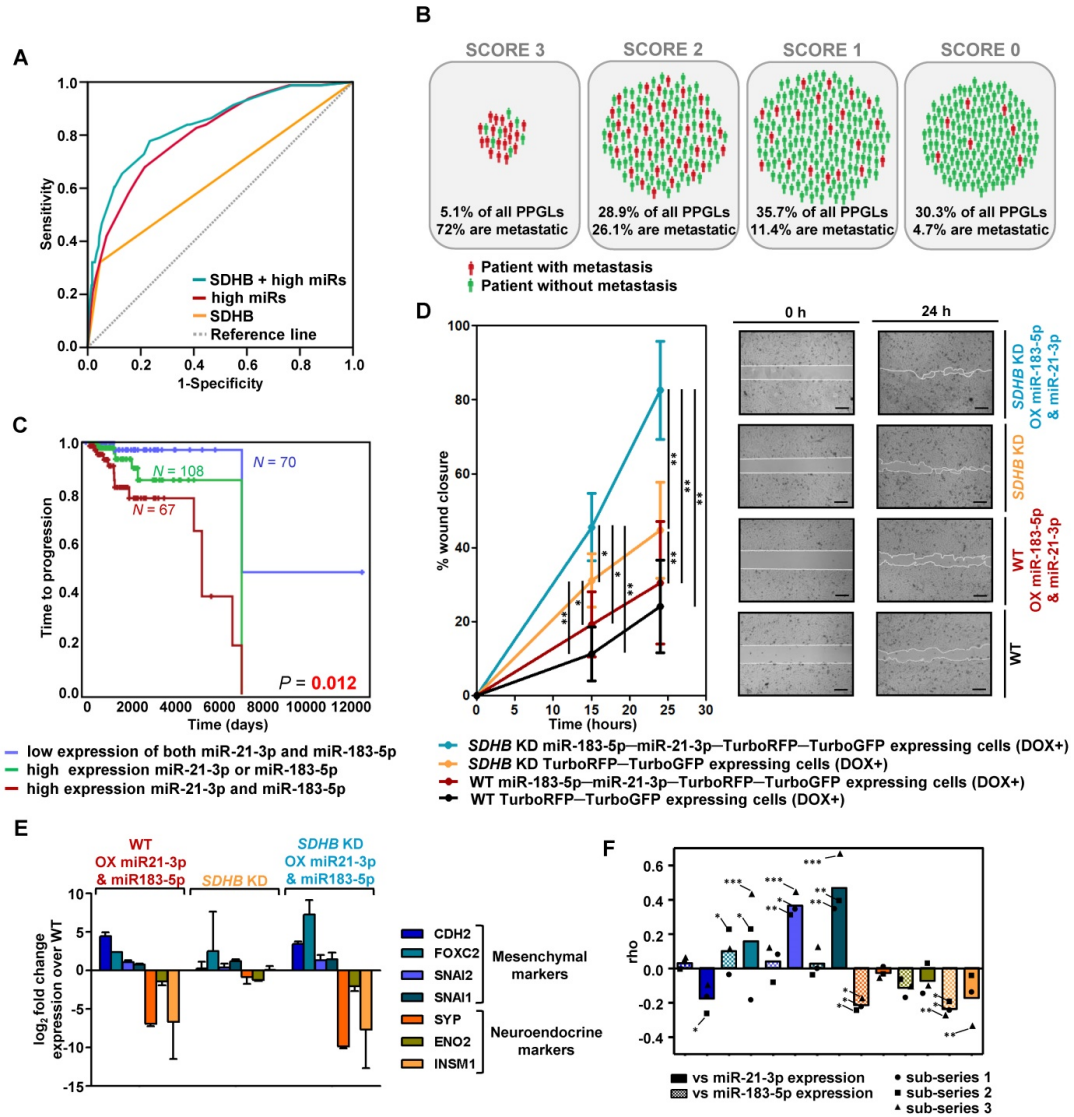
** Risk model of metastasis prediction. (A)** Receiver operating characteristic curve analysis showing the accuracy of the miR-*SDHB* classifier to discriminate mPPGL patients. Data correspond to all samples from the discovery plus the validation series (*n*=492). miRNAs associated with a shorter TTP were included in the analysis. To combine the data of the different series for the generation of the model, the expression of each miRNA was expressed as a dichotomous variable using the median miRNA expression as the cutoff in each series. Binary variables that identified the different series and the *SDHB* status were included. AUC, 95%CI and P-values are given in the text. **(B)** Schematic representation of patients from discovery and validation series (*n*=492) divided into four groups depending on the miR-*SDHB* classifier (score 3 = high miR-183-5p and high miR-21-3p and *SDHB* mutated; score 2 = high miR-21-3p or high miR-183-5p and *SDHB* mutated, or high miR-21-3p and miR-183-5p and *SDHB* not mutated; score 1 = high miR-21-3p or high miR-183-5p or *SDHB* mutated; score 0 = none of the aforementioned criteria apply). Red icons: patients with mPPGL; green icons: non-metastatic patients. **(C)** Kaplan-Meier plot of TTP of patients according to the expression levels of the miRNAs indicated. High and low expression indicates expression above and below the median expression level of the whole group, respectively. *N*: number of patients. *P*-values were calculated with a log-rank test. **(D)** Wound healing assay in SK-N-AS cells with/without *SDHB* stably silenced and with/without ectopic expression of miR-21-3p and miR-183-5p. Wound area was assessed at 0, 15 and 24 h. Quantification is based on 18 pictures/condition, *n*=2. Error bars represent SD.^*^P<0.05, ^**^P<0.01; two-tailed unpaired t-test. Scale bars=100 µm. **(E, F)** Expression of the indicated mesenchymal and neuroendocrine genes observed in the SK-N-AS cell model and in the discovery series. **(E)** Log_2_ fold change expression of the indicated genes relative to WT TurboRFP-TurboGFP-expressing control cells after normalization of each sample to β-actin. All cells were pretreated with doxycycline (1µg/ml, 120h). Expression is reported as mean of triplicates and error bars represent SD. **(F)** Spearman's correlations (rho) between the genes shown in Figure (E) and miR-21-3p (smooth columns) or miR-183-5p (dotted columns) expression in the discovery series. Bars indicate the mean of the 3 sub-series; individual values of each sub-series are also shown (•=sub-series 1, ■=sub-series 2, ▲=sub-series 3). ^***^P<1·10^-9^, ^**^P<1·10^-4^, ^*^P<0.05. DOX+: doxycycline pretreated cells (1µg/ml, 120h). WT: wild type; OX: overexpressing; KD: knock down.

With the validated miRNAs associated with shorter TTP, we established a prognostic predictive model. The final model, corrected for *SDHB* status, selected miR-21-3p and miR-183-5p as the best predictive markers of metastases. The miRNA-based classifier (AUC=0.804, 95%CI=0.753-0.855, *P*=4.67·10^-18^) showed higher accuracy than *SDHB* genotype alone (AUC=0.637, 95%CI=0.563-0.711, *P*=9.29·10^-5^), while the predictive value increased when adding *SDHB* status to the model (AUC=0.837, 95%CI=0.787-0.886, *P*=9.88·10^-22^) (Figure 2A). When segregating patients according to their classifier features, 72% of score 3 (high miR-183-5p and miR-21-3p levels; *SDHB* mutated) patients were metastatic, compared to the 4.7% of score 0 (low miR-183-5p and miR-21-3p expression, and no-*SDHB* mutated) (Figure 2B). Concurrent high expression of miR-21-3p and miR-183-5p was significantly more associated with shorter TTP than high expression of either miRNA alone (Figure 2C).

### High miR-183-5p and miR-21-3p expression trigger features related to a pro-metastatic phenotype

To further explore the role of miR-183-5p and miR-21-3p in metastasis, we evaluated in a cell model migration, proliferation and transition to a mesenchymal state, characteristics classically associated with an aggressive phenotype.

*SDHB*-deficient cells have an increased migration capacity compared to WT cells 29. We replicated this phenotype in a wound healing assay and also observed increased migration of cells overexpressing miR-183-5p and miR-21-3p, both in the presence and absence of a functional *SDHB* gene (Figure 2D). However, we did not observe differences in cell proliferation.

It has been reported that *SDHB* loss is associated with a neuroendoMT 30, and we therefore assessed the expression of neuroendocrine (*SYP*, *ENO2*, *INSM1*) and mesenchymal (*CDH2*, *FOXC2*, *SNAI2*, *SNAI1*) markers to study whether overexpression of miR-21-3p and miR-183-5p acerbated this feature. Although cells did not show obvious morphological changes, overexpression of miR-21-3p and miR-183-5p increased expression of mesenchymal markers and decreased expression of neuroendocrine ones, both in WT and in *SDHB*-silenced cells (Figure 2E).

Expression of the same markers was evaluated in the discovery series. We observed that mesenchymal markers showed a positive correlation and neuroendocrine markers a negative one with the expression of the two miRNAs (Figure 2F). miR-21-3p generally increased the expression of mesenchymal markers, whereas miR-183-5p decreased the expression of neuroendocrine genes.

### Integration of mRNA and miRNA expression reveals that *TSC2* mRNA is reduced upon high miR-21-3p levels

**Figure 3:**
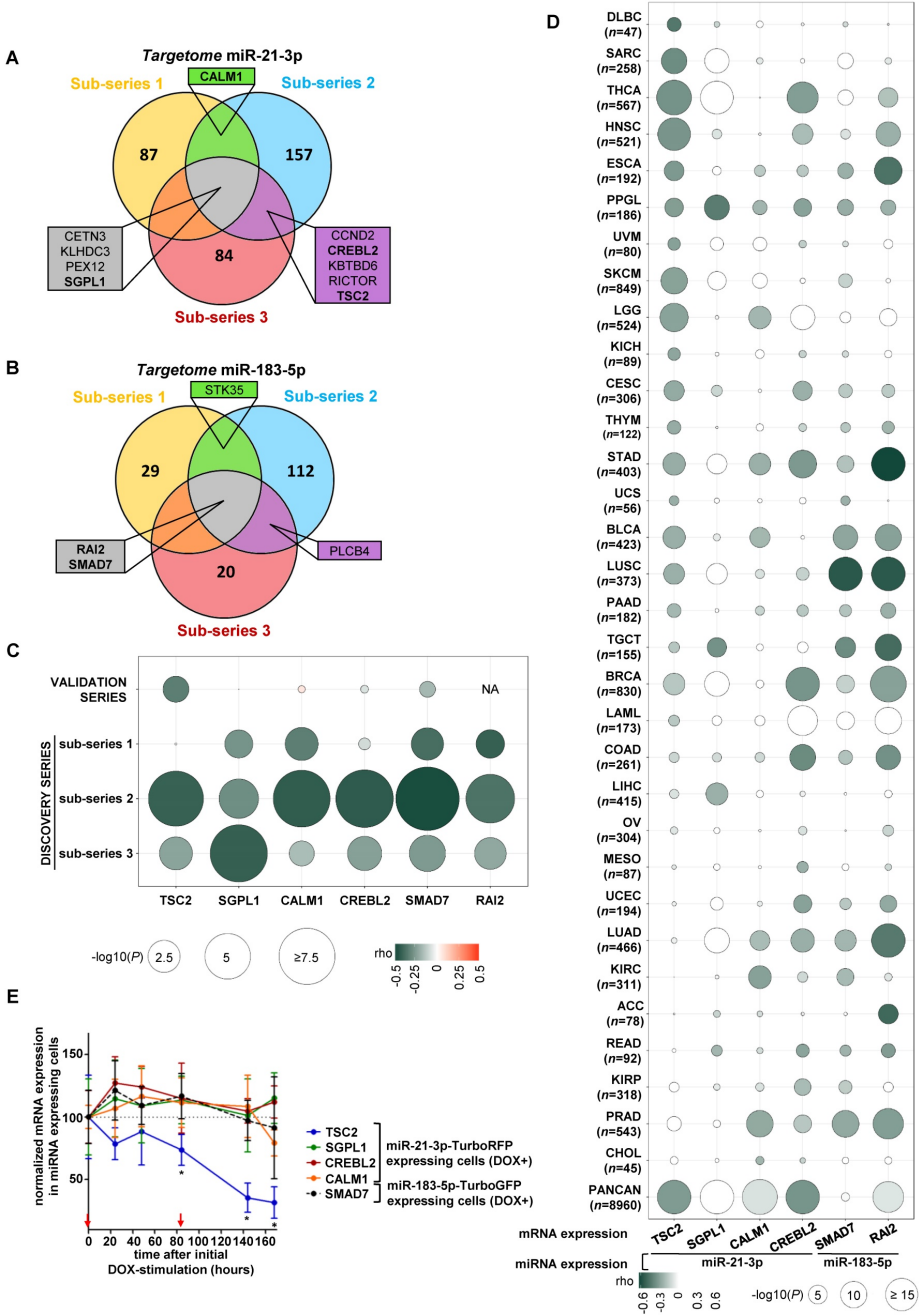
** Identification of potential gene targets of miR-21-3p and miR-183-5p. (A, B)** Venn diagrams summarizing significant genes showing a negative correlation (*P*<0.05) with miR-21-3p and miR-183-5p expression, respectively, in each sub-series of the Discovery series. Only genes shared between at least two sub-series are shown; rho and p-values are shown in Tables S4 and S5. Genes selected for validation are shown in bold. **(C)** Bubble diagram showing the correlation between mRNA levels of selected target genes in the discovery and validation series; the colors of the bubbles indicate the rho-value and their diameter is proportional to - log_10_(*P*) as indicated below the panel. NA: data not available. **(D)** Bubble diagrams showing the correlations between mRNA expression of the selected target genes and miRNA expression across 32 TCGA projects representing the major cancer types: LAML, acute myeloid leukemia; ACC, adrenocortical carcinoma; BLCA, bladder urothelial carcinoma; LGG, brain lower grade glioma; BRCA, breast invasive carcinoma; CESC, cervical squamous cell carcinoma and endocervical adenocarcinoma; CHOL, cholangiocarcinoma; COAD, colon adenocarcinoma; READ, rectum adenocarcinoma; ESCA, esophageal carcinoma; HNSC, head and neck squamous cell carcinoma; KICH, kidney chromophobe; KIRC, kidney renal clear cell carcinoma; KIRP, kidney renal papillary cell carcinoma; LIHC, liver hepatocellular carcinoma; LUAD, lung adenocarcinoma; LUSC, lung squamous cell carcinoma; DLBC, lymphoid neoplasm diffuse large B-cell lymphoma; MESO, mesothelioma; OV, ovarian serous cystadenocarcinoma; PAAD, pancreatic adenocarcinoma; PCPG, pheochromocytoma and paraganglioma ('PPGL' in our report); PRAD, prostate adenocarcinoma; SARC, sarcoma; SKCM, skin cutaneous melanoma; STAD, stomach adenocarcinoma; TGCT, testicular germ cell tumors; THYM, thymoma; THCA, thyroid carcinoma; UCS, uterine carcinosarcoma; UCEC, uterine corpus endometrial carcinoma; PANCAN: dataset downloaded from UCSC Xena. Glioblastoma multiforme was not included in the study since miRNA expression data was not available in UCSC Xena. n: number of samples per project. Colors and diameters of the bubbles are as in (C) and are summarized at the bottom of the panel. **(E)** Normalized mRNA expression of genes selected from the targetome in miR-21-3p─TurboRFP expressing cells (*TSC2*, *SGPL1*, *CREBL1*, *CALM1*) or miR-183- 5p─TurboGFP expressing cells (*SMAD7*). Expression for each condition is normalized to β-actin and expression in control cells (mean + SD; *n*=3). We represent the average of WT and *SDHB* KD cells of the mean-centered expression of the miRNA expressing cells (DOX+) over TurboRFP or TurboGFP expressing control cells (DOX+), respectively after initial doxycycline stimulation (1µg/ml) at t0. Two-tailed unpaired t-test was applied to test for differences with control cells not overexpressing miRNA (*: *P*<0.05). Red arrows indicate medium changes (DOX-stimulation).

We integrated the miR-21-3p and miR-183-5p expression data with their *targetomes* to identify potential relevant targets for these miRNAs. A significant negative correlation was observed in 43.3% of predicted miR-21-3p targets, and in 10.7% of miR-183-5p ones in at least one sub-series (Figure 3A and 3B); of these, only ten genes met the established filtering criteria for miR-21-3p and four for miR-183-5p (Table S5 and S6). After applying biological criteria (Table S7), six genes (*TSC2*, *SGPL1*, *CREBL2* and *CALM1* for miR-21-3p; *SMAD7* and *RAI2* for miR-183-5p) were selected for replication in the validation series (Figure 3C). Only *TSC2* expression showed a significant negative correlation with miR-21-3p expression in this series (ρ=-0.324, *P*=0.023).

In parallel, 32 cancers TCGA data sets were used to assess the correlation between the expression of selected gene-miRNA pairs. Of them, 62.5% showed a significant negative correlation between miR-21-3p and *TSC2* expression. Furthermore, the analysis performed with the whole PanCan (*n*=8,960 tumors) cohort indicated that only *TSC2* and *CREBL2* showed a significant negative correlation (ρ<-0.3, *P*<1·10^-200^) with miR-21-3p expression (Figure 3D).

Expression of the six aforementioned genes was evaluated in the cell models overexpressing the miRNAs, and only *TSC2* showed a significant decrease in expression (Figure 3E).

Using TargetScan (http://www.targetscan.org/vert_72/), we found binding sites at 3'UTR in *CREBL2, SGPL1* and* CALM1* (Figure S3A, S3B and S3C) for miR-21-3p, but none in the remaining genes. However, extensive visual inspection of miRNA sequences and the 3'UTRs of *TSC2*, *SMAD7* and *RAI2* revealed potential binding sites (Figure S3A, S3E and S3F).

### Role of miR-21-3p in mTOR pathway regulation

**Figure 4:**
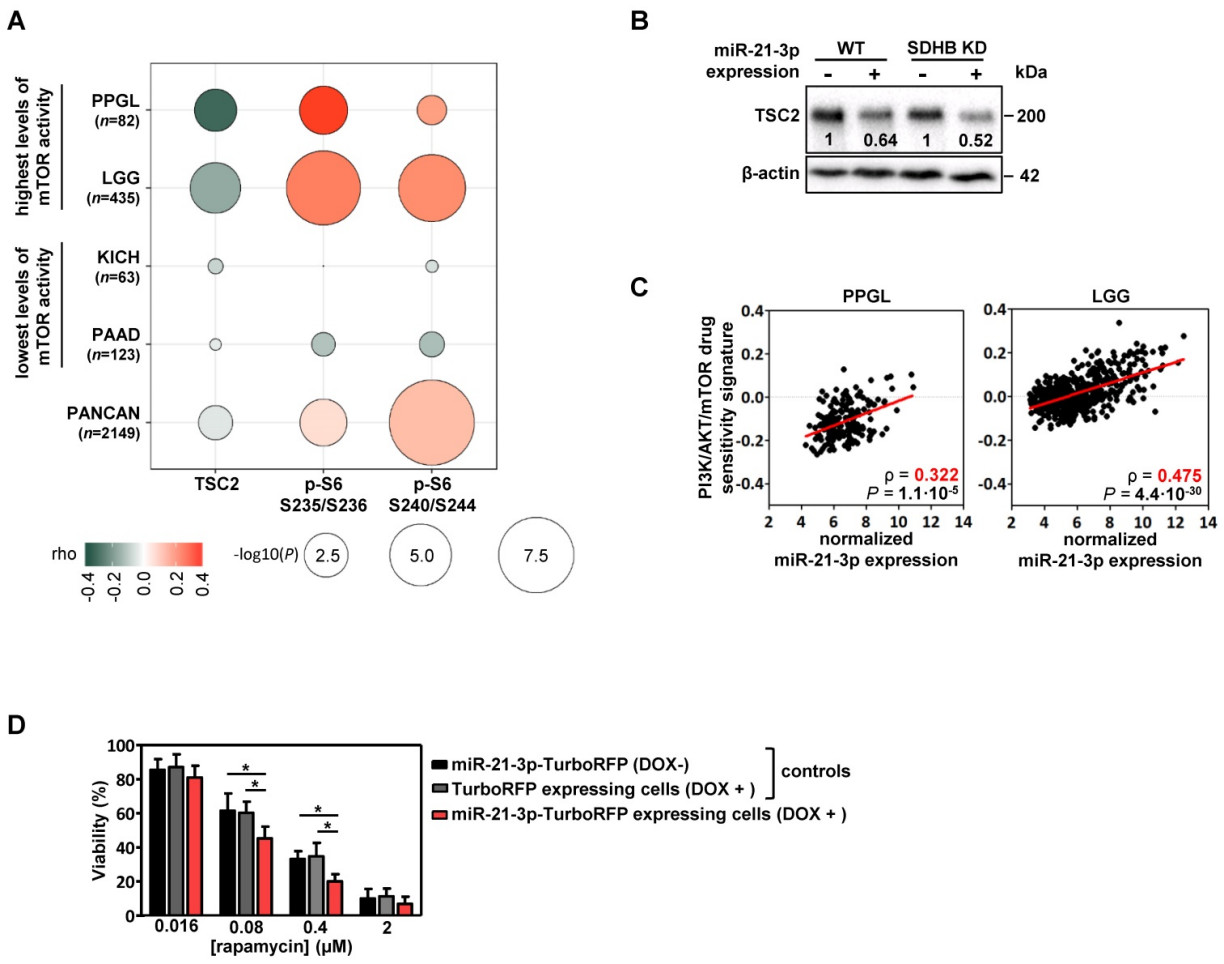
** Involvement of miR-21-3p in mTOR pathway regulation. (A)** Bubble plot indicating Spearman's correlations (rho) between key mTOR pathway proteins that could be affected by *TSC2* mRNA downregulation and miR-21- 3p upregulation. TCGA RPPA data of TSC2 protein, p-S6 S235/236 and S240/244 was correlated with miR-21-3p expression in PPGL and LGG TCGA samples (showing the highest mTOR pathway activity reported by Zhang *et al.*^25^), as well as in KICH and PAAD samples (lowest levels of mTOR pathway activity); bubble colors indicate the rho coefficient and their diameter is proportional to - log_10_(*P*) as indicated below the panel. **(B)** Representative western blot of TSC2 expression in SK-N-AS WT and *SDHB* KD cells, with or without ectopic expression of miR-21-3p (miR-21-3p-TurboRFP expressing cells DOX + and control TurboRFP expressing cells DOX +, respectively). Relative quantification of the TSC2 signal in miR-21-3p expressing cells was normalized to the one in TurboRFP expressing cells (DOX +). B-actin has been used as loading control.** (C)** Scatter plots showing the correlation between miR-21-3p expression and PI3K/AKT/mTOR drug sensitivity signature (from Zhang *et al.*^25^) in PPGL (n=178) and LGG (n=514) tumors from the TCGA project. Spearman's correlation coefficient (ρ) and *P*-values are shown. **(D)** Cell viability percentage in SK-N-AS miR-21-3p - TurboRFP cells (induced with doxycycline [DOX+] or not [DOX-]) and in SK-N-AS TurboRFP [DOX+] cells treated with the indicated concentrations of rapamycin for 96h. Proportion of viability is shown as the mean ± SD of 2-paired independent experiments performed in triplicate. Two-tailed unpaired t-test was applied to test for differences (*: *P*<0.01). DOX+: doxycycline pretreated cells (1µg/ml, 120h).

Using functional proteomics data from the TCGA project, we evaluated the effect that miR-21-3p could have on protein levels. In PPGLs, we found a significant negative correlation for miR-21-3p expression with TSC2 protein levels (ρ=-0.31, *P*=4.5·10^-3^), as well as a positive one with pS6 (in Ser235/236: ρ=0.36, *P*=9.1·10^-4^; in Ser240/244: ρ=0.20, *P*=0.07) (Figure 4A); a positive correlation tendency with pAKT levels was also observed (Figure S4).

We also explored the potential role of miR-21-3p in other cancer types with high levels of mTOR activity 25. Apart from PPGL, low-grade glioma (LGG) also exhibits a significant negative correlation between miR-21-3p and TSC2 (ρ=-0.18, *P*=1.3·10^-4^), as well as a positive one with pS6 levels (in Ser235/236: ρ=0.27, *P*=1.7·10^-8^; in Ser240/244: ρ=0.23, *P*=6.6·10^-7^) (Figure 4A). By contrast, in cancer types with the lowest levels of mTOR activity (kidney chromophobe -KICH- and pancreatic adenocarcinoma -PAAD-) no significant correlations were observed. Downregulation of TSC2 in cells overexpressing miR-21-3p was further demonstrated by Western blot (Figure 4B).

To determine whether miR-21-3p levels could also be associated with an increased response to drugs targeting mTOR pathway, we explored the inhibitor-sensitivity mRNA signature proposed by Zhang *et al.* 25 for TCGA project samples. This signature score correlated significantly with miR-21-3p levels in PPGL and LGG (Figure 4C), but not for KICH and PAAD (Figure S5).

Furthermore, cells with ectopic expression of miR-21-3p showed a significant reduction in cell proliferation after rapamycin treatment in comparison to their controls (Figure 4D).

### Metastatic miRNA signature is detected in the circulation

**Figure 5:**
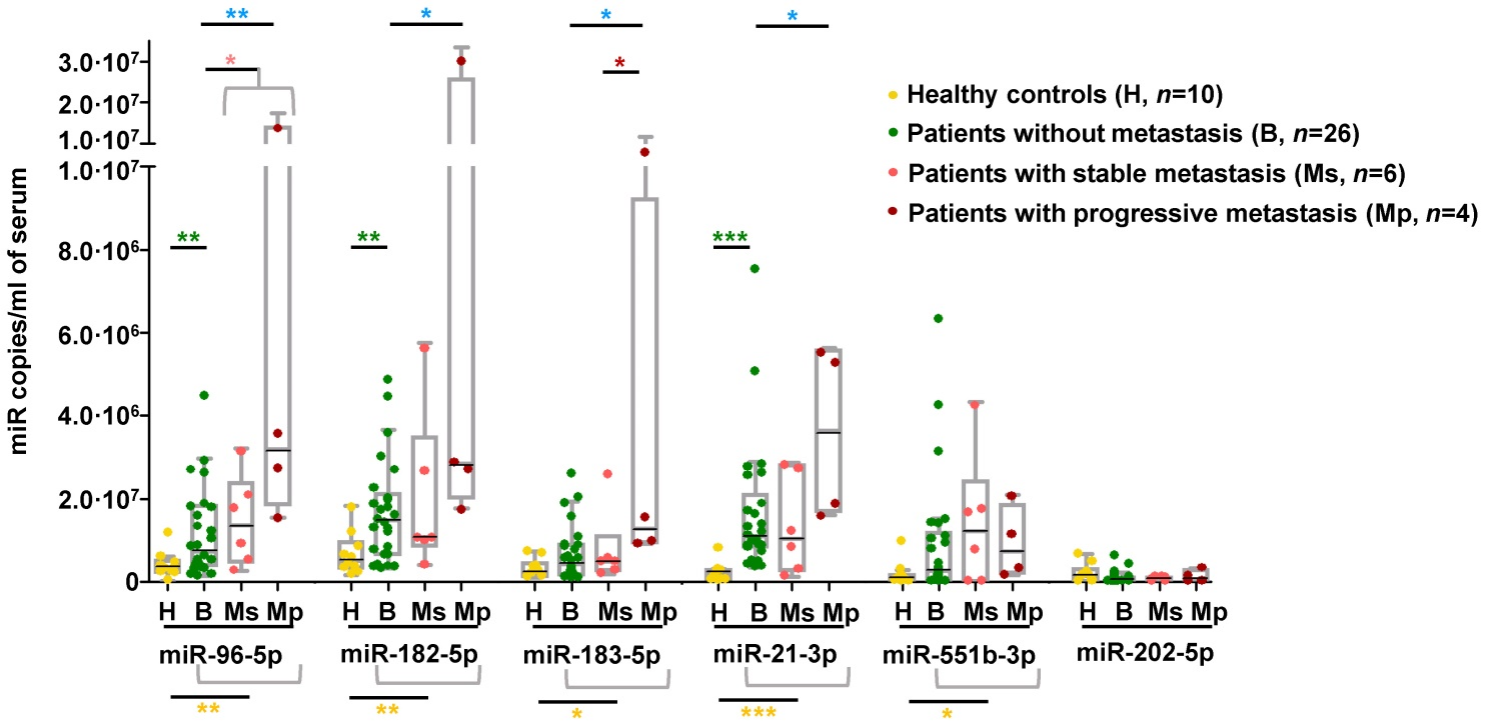
** Detection of mPPGL-related miRNAs in the circulation.** Box and whisker plots indicate serum levels of the indicated miRNAs in healthy (H, yellow), non-metastatic (B, green) and mPPGL patients (Ms and Mp; orange and brown, respectively) determined by ddPCR. Distinction between stable mPPGL (Ms) and progressive mPPGL (Mp) was based on clinical and radiological data (see Table S8). Mann-Whitney test was applied to test for significant differences. Significant differences between H and the patient cohort (including B, Ms and Mp) are indicated with yellow asterisks, between H and B with green asterisks, between B and metastatic (including Ms and Mp) patients with pink asterisks, between B and Mp with blue asterisks, and Ms and Mp with red asterisks. ^*^: *P*<0.05, ^**^: *P*<0.01 and ^***^: *P*<0.00001.

We explored whether the miRNA signature displayed by metastatic tumors could be detected in circulation of an independent series of PPGL patients. We observed that serum levels of all miRNAs of the signature except miR-202-5p were significantly higher in PPGL patients than in healthy controls, regardless of the metastatic status (Figure 5). miR-96-5p, miR-182-5p and miR-21-3p remained elevated in PPGL vs. healthy individuals, with miR-21-3p being the best discriminator (AUC=0.96; *P*=0.0001). Notably, circulating levels of all miRNAs of the signature (except miR-551b-3p and miR-202-5p) exhibited a tendency towards increased expression in mPPGLs compared to non-metastatic cases, reaching statistical significance in the mPPGL subgroup with evidence of progressive disease (Table S8). Levels of miR-183-5p remained significantly higher in patients with progressive vs. stable mPPGL. Levels of miR-21-3p were significantly correlated with metastatic burden (r=0.38; *P*=0.04).

To better ascertain if miRNAs of the metastatic signature are secreted by chromaffin cells, we quantified levels of these miRNAs in conditioned medium of imCCs, a model of PPGL. Consistent with the findings in serum of PPGL patients, miR-202-5p and miR-551b-3p, which exhibited low levels in patients, were undetectable in imCCs, whereas the levels of all other miRNAs were higher in *Sdhb*-/- compared to WT cells (Figure S6).

## Discussion

Precision cancer medicine focuses on patients' stratification according to their metastatic risk, and/or potential treatment benefits. Rare cancers are becoming models for personalized medicine as more clinical, genetic and genomic knowledge leads to insights into signaling pathways and mechanisms underlying their pathogenesis. Here, we propose a new metastatic risk miRNA signature, with prognostic value and related to shorter TTP. Eventually, this signature could be measured as a circulating biomarker in serum from PPGL patients. Furthermore, our study reveals a new putative miR-21-3p/TSC2/mTOR regulatory axis as a potential treatment target.

The high expression cluster miR-183/96/182-5p in mPPGL was already reported in SDHx-related PPGLs 16, 17, but we now identified its high expression in mPPGLs regardless of their genotype. We also uncovered three novel markers of mPPGL, which have been described to be deregulated in multiple cancers (see Table S4). The fact that the model only includes miR-21-3p and miR-183-5p, and is able to discriminate metastatic patients with higher accuracy than *SDHB*-positive status (widely accepted as a mPPGL marker), suggests that additional important molecular features are involved in PPGL progression. This hypothesis is supported by the enhanced collective cell migration, likely driven by a neuroendoMT phenotype, observed in the wound healing assay after overexpression of miR-21-3p and miR-183-5p in an *SDHB*-silenced cell line.

The integration of miR-21-3p and miR-183-5p with mRNA data identified targets potentially regulated by those miRNAs, and that miR-21-3p may be involved in the regulation of the mTOR pathway through *TSC2* mRNA downregulation. In this regard, the 3' UTR of *TSC2* contains a 6mer pairing with miR-21-3p with a single nucleotide mismatch, which is compensated by a 4nt compensatory site downstream of the miRNA pairing sequence, similar to previously reported cases 31. In addition, although other mechanisms such as targeting the 5'UTR and the open reading frame could also be considered, we cannot exclude the possibility of indirect effects of other responsive UTR targets of miR-21-3p as 43.3% of the 852 potential miR-21-3p gene targets showed an inverse correlation with the miRNA levels. In fact, we also found a weaker positive correlation of miR-21-3p with pAKT levels in PPGL and LGG, indicating a possible regulation upstream of the pathway through AKT phosphorylation, TSC1-TSC2 complex destabilization, TSC2 ubiquitination and, consequently, mTOR pathway activation 32.

To note, constitutive activation of PI3K/AKT/mTOR signaling is a feature described in PPGLs and associated with increased activation of pAKT 33. Actually, both PPGL and LGG exhibit activation of this pathway despite a lack of canonical somatic alterations 25. Taking advantage of the phospho-proteomics data from the TCGA project, we have shown that miR-21-3p correlates significantly with markers of mTOR pathway activation. We did not observe the effects on mTOR pathway activation after miR-21-3p overexpression in the cell model. However, protein output can vary by 2-fold without detectable effects, and consequences of most of the miRNAs alone are challenging to detect in the lab 31. Taking into account our findings, additional mechanisms of activation of the mTOR pathway, such as miR-21-3p regulation, should be considered.

Studies targeting mTOR in preclinical PPGL models have provided some promising results 34-38, and there is also immunohistochemical evidence of heterogeneous mTOR activation in mPPGLs 39-41. Nevertheless, discordant results have been reported in clinical studies with everolimus 42-44 that could be due to the absence of appropriate inclusion criteria using molecular markers. In fact, few studies have examined markers for stratifying mPPGL patients for targeted molecular therapies 3. With this in mind, we have found in PPGL and LGG that miR-21-3p shows a significant correlation with a sensitivity signature of drugs targeting mTOR pathway. The enhanced sensitivity to rapamycin of our miR-21-3p overexpressing cell model indicates that, indeed, miR-21-3p could be a potential marker for selecting candidate patients for treatment with mTOR inhibitors.

Our findings support the concept of a miRNA signature for patient stratification according to metastatic risk**.** Importantly, a significant fraction of the metastatic miRNA signature was detected in the serum of an independent cohort of patients showing increased levels of these miRNAs. miRNAs associated with a shorter TTP in tumor tissue (miR-21-3p, miR-183-5p, miR-182-5p and miR-96-5p) were also increased in the serum of metastatic cases and showed the highest levels during the progressive phase of metastasis. The mechanisms of how these metastasis-related miRNAs enter the circulation and whether they are biologically functional warrant further investigation. We show that circulating miRNAs are indeed secreted by imCCs, and that this secretion is increased in cells with a more aggressive phenotype 30. While these miRNAs may have cell-intrinsic roles, as we showed for miR-21-3p, it is also possible that they participate in cell-cell communication by controlling the tumor microenvironment. Regulation of gene expression by miRNAs outside the cell of origin may also account for cancer progression and therapy resistance 45. These findings extend the potential clinical utility of detecting the metastatic miRNA signature in liquid biopsies, but further prospective studies will be required to address this issue.

In conclusion, the power of a large sample series combined with multi-omic integration and the accessibility of TCGA data of other cancer entities has enabled the identification of robust biomarkers relevant to disease progression. This strategy is also a powerful tool for elucidating appropriate therapeutic options based on molecular biomarkers in PPGL, which is a significant step towards achieving precision medicine for patients with mPPGL - a disease with no FDA-approved therapies 2.

## Supplementary Material

Supplementary methods, figures and tables.

## Abbreviations

PPGL: Pheochromocytoma and paraganglioma
mPPGL: metastatic pheochromocytoma and paraganglioma
TTP: time to progression
ROC: receiver operating characteristic
AUC: area under the ROC curves
FBS: fetal bovine serum
neuroendoMT: neuroendocrine-to-mesenchymal transition
qPCR: quantitative real-time PCR
CM: conditioned media
SF: serum-free
WT: wild-type
SDHB KD: SDHB knocked down
TCGA: The Cancer Genome Atlas
LGG: low-grade glioma
KICH: kidney chromophobe
PAAD: pancreatic adenocarcinoma
imCCs: immortalized mouse chromaffin cells
