# Supplementary material for: Integrative multi-omics analysis identifies a prognostic miRNA signature and a targetable miR-21-3p/TSC2/mTOR axis in metastatic pheochromocytoma/paraganglioma
Source: Theranostics. 2019 Jul 9;9(17):4946–58. doi: 10.7150/thno.35458 (PMC6691382; doi:10.7150/thno.35458)
Supplement: Supplementary file 1 — Supplementary methods, figures and tables. [file thnov09p4946s1.pdf]

SUPPLEMENTAL INFORMATION

Integrative multi-omics analysis identifies a prognostic miRNA signature and a targetable miR-21-3p/TSC2/mTOR axis in metastatic pheochromocytoma/paraganglioma

Bruna Calsina, Luis Jaime Castro-Vega, Rafael Torres-Pérez, Lucía Inglada-Pérez, Maria Currás-Freixes, Juan María Roldán-Romero, Veronika Mancikova, Rocío Letón, Laura Remacha, María Santos, Nelly Burnichon, Charlotte Lussey-Lepoutre, Elena Rapizzi, Osvaldo Graña, Cristina Álvarez-Escolá, Aguirre A de Cubas, Javier Lanillos, Alfonso Cordero-Barreal, Ángel M Martínez-Montes, Alexandre Bellucci, Laurence Amar, Fabio Luiz Fernandes-Rosa, María Calatayud, Javier Aller, Cristina Lamas, Júlia Sastre-Marcos, Letizia Canu, Esther Korpershoek, Henri J Timmers, Jacques WM Lenders, Felix Beuschlein, Martin Fassnacht-Capeller, Graeme Eisenhofer, Massimo Mannelli, Fátima Al-Shahrour, Judith Favier, Cristina Rodríguez-Antona, Alberto Cascón, Cristina Montero-Conde, Anne-Paule Gimenez-Roqueplo, Mercedes Robledo

1) Supplementary Figures S1-S6

2) Supplementary Tables S1-S8

3) Supplementary Methods

Figure S1

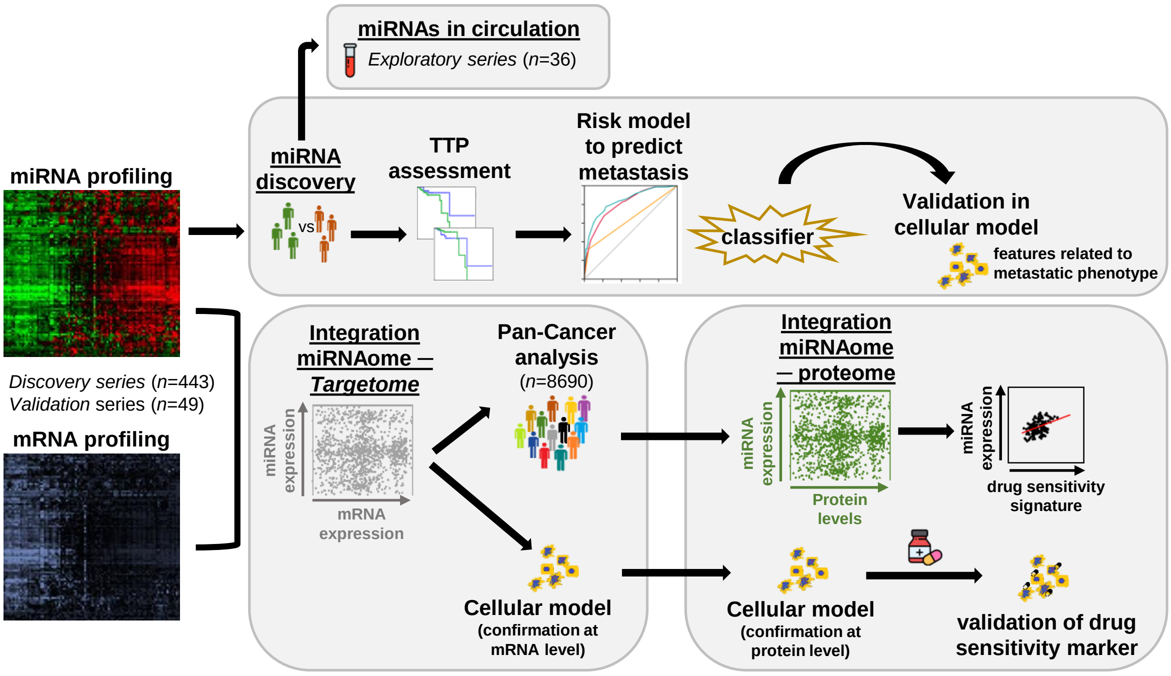

Figure S1. Step-by-step scheme summarizing the procedure of the present study.
Each step is described in greater detail in the Methods section.

Figure S2

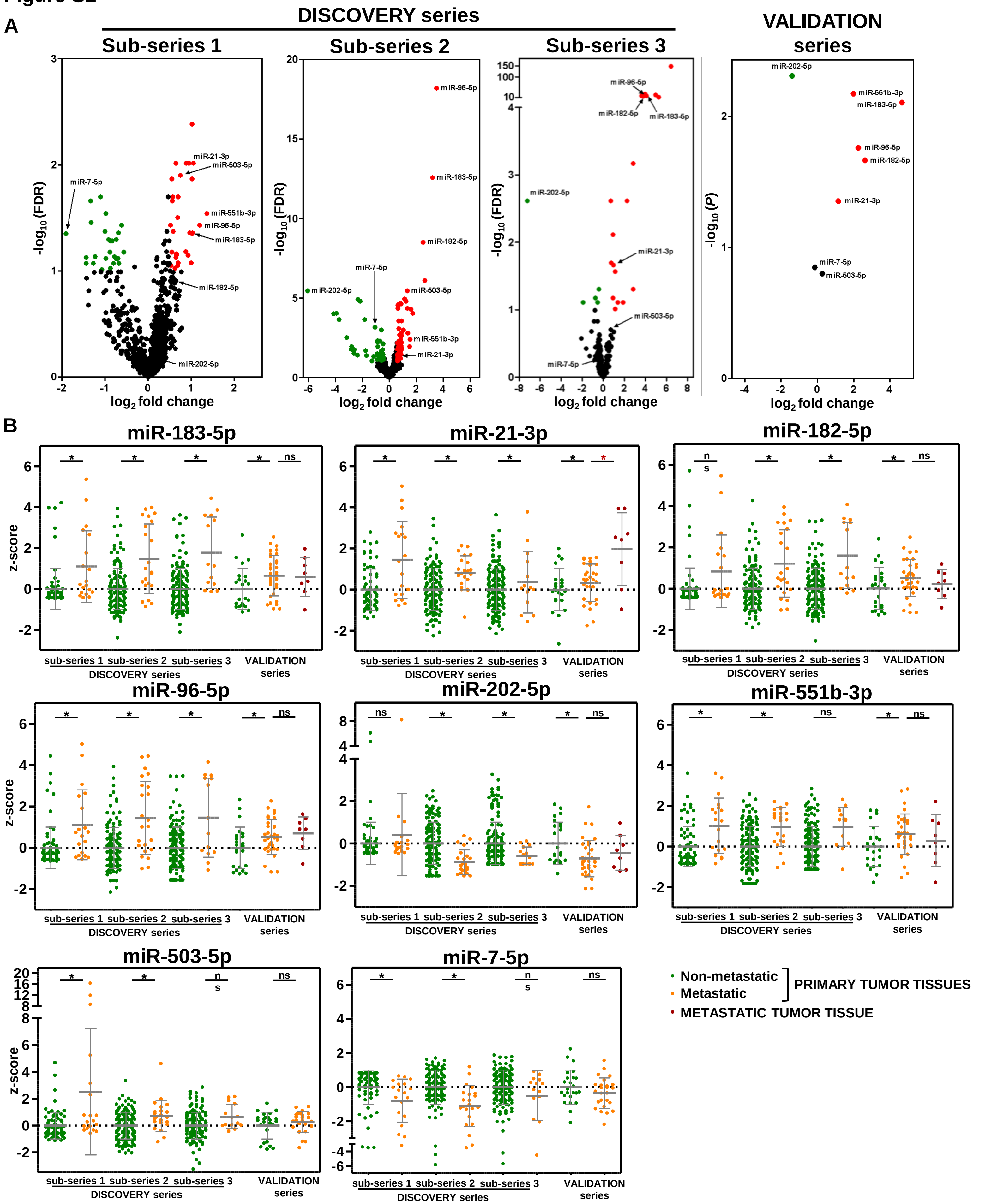

Figure S2. **miRNAs associated with metastatic behavior**.

(A) Volcano plot analysis of miRNAs in the different series. The x-axis shows the $\log_2$ fold change of miRNA expression, whereas the y-axis shows the $-\log_{10}$ (FDR) or $-\log_{10}$ (P) for each miRNA. MiRNAs with $P<0.05$ are colored to indicate whether they are up-regulated (red) or down-regulated (green) in tumors of patients with mPPGL. miRNAs selected for validation are indicated.

(B) Expression of miRNAs selected for validation in the different series. Log_2-normalized expression from the different series is displayed as a transformed z-score (centered at the mean of the non-metastatic group for each series). miRNA expression in primary tumors of patients with non-mPPGL is shown in green, in primary tumors of patients with mPPGL in orange, and in metastatic tissues in red (metastatic tissues were only available from 8 patients, whose paired primary tumors are included in the orange group and the expression was only evaluated for the validated miRNAs). Data are shown as mean ± SD. * *FDR*<0.05 for the discovery series obtained during the differential expression analysis, and *P*<0.05 from a nonparametric Mann-Whitney test in the validation series.

Figure S3

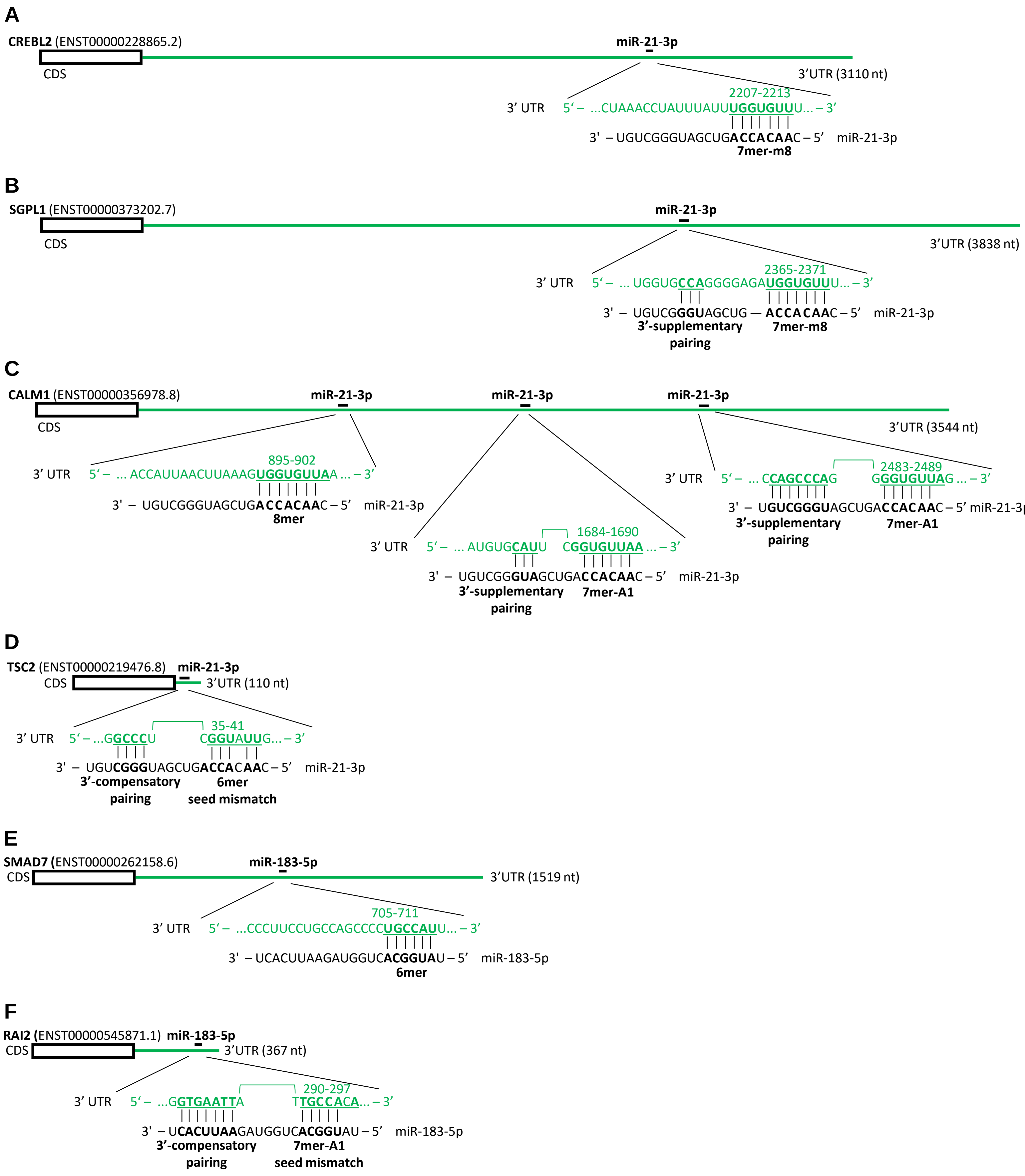

Figure S3. Sequence of the 3'-UTRs of selected potential target genes of miR-21-3p and miR-183-5p. The close-ups show miRNA annealed to the target sequence (in bold). Binding sites are shown for miR-21-3p in (A) *CREBL2*, (B) *SGPL1*, (C) *CALM1* and (D) *TSC2*; and for miR-183-5p in (E) *SMAD7* and (F) *RAI2*.

Figure S4

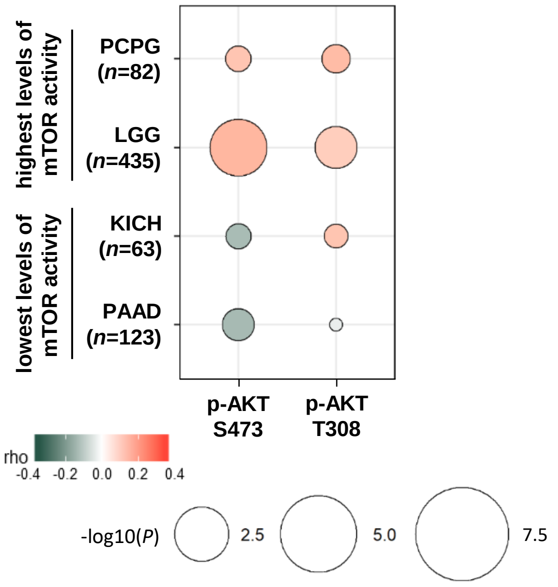

Figure S4. **Bubble plot indicating Spearman's correlations (rho) between pAKT and miR-21-3p expression.** TCGA RPPA data of p-AKT S473 and T308 was correlated with miR-21-3p expression in PPGL and LGG TCGA samples (highest mTOR pathway activity reported by Zhang *et al*. [25]), as well as in KICH and PAAD samples (lowest levels of mTOR pathway activity).

Figure S5

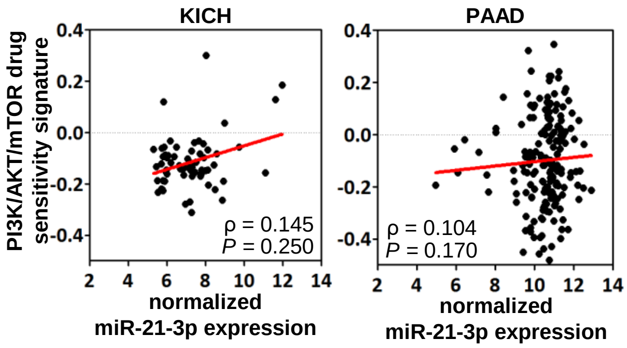

Figure S5. **Scatter plots showing the correlation between miR-21-3p expression and PI3K/AKT/mTOR drug sensitivity signature (from Zhang *et al*.[25]) in KICH (*n*=65) and PAAD (*n*=177) tumors from the TCGA project.** Spearman's correlation coefficient (ρ) and *P* are shown.

Figure S6

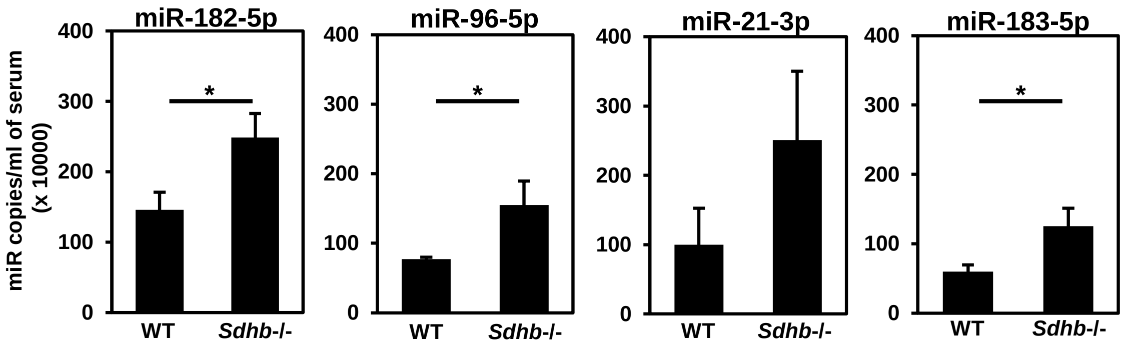

Figure S6. Levels of miRNAs in conditioned medium of wild type (WT) and Sdhb-/- immortalized mouse chromaffin cells (imCCs). Plots show levels of the indicated miRNAs determined by ddPCR in conditioned medium preparations (cells incubated for 24h in serum-free media) equivalent to 60,000 cells. Levels of miR-202-5p and miR-551b-3p were undetectable. Error bars indicate standard error of the mean from three independent conditioned medium preparations. Unpaired t-test was applied to test for differences (*: P<0.05).

Table S1. Clinical data of patients included in the validation series and patients with primary-metastatic paired samples available

		**METASTATIC**<br>– 25 cases –	**BENIGN** (>4 yr. follow-up)<br>– 24 cases –	**PRIMARY-METASTATIC PAIRED SAMPLES**<br>– 8 cases –
**genotype**	*SDHB*	7 (28%)	2 (8%)	6
	SDHD	3	-	-
	NF1	2	1	1
	VHL	2	3	-
	RET	2	5	-
	SDHA	1	1	-
	HRAS	1	2	-
	EPAS1	1	1	-
	MAX	-	1	-
	ATRX	1	-	-
	MDH2	1	-	-
	GOT2	1	-	-
	WT	3	8	1
**location**	PGL	12	4	6
	PCC	8	15	2
	PGL & PCC	5	5	-

For the validation series, twenty-four cases were classified as non-metastatic, as patients were disease free at the time of the last clinical follow-up (≥ 4 years follow-up, median=8.5 years). The remaining twenty-five samples were classified as metastatic since the patients presented tumor cells at non-chromaffin sites. The proportion of *SDHB* cases per group (28% in the metastatic and 8% in the non-metastatic group) was similar to that observed in the discovery series (average in the three sub-series of 24.3% and 4.4% in metastatic and non-metastatic groups, respectively). WT: wild type; PGL: Paraganglioma; PCC: Pheochromocytoma.

Table S2. Clinical data of patients included in the exploratory series of serum samples

**PPGL patients**	**(*n*)**
Male	23
Female	13
Mean age at diagnosis (range)	37.2 years (13-64)
Pheochromocytoma	21
Paraganglioma	15
SDHA	2
SDHB	6
SDHD	3
VHL	11
NF1	3
RET	2
HRAS	2
MAX	1
Sporadic	6
Metastatic	10
Non-metastatic	26

**Healthy individuals**	**(*n*)**
Male	6
Female	4
Mean age (range)	32.8 years (23-43)

Table S3. Differentially expressed miRNAs in metastatic vs. non-metastatic tumors in at least one sub-series of the *Discovery* cohort

	Discovery series						Validation series	
	Sub-series 1		Sub-series 2		Sub-series 3			
	log2FC	FDR	log2FC	FDR	log2FC	FDR	log2FC	p-value
hsa-miR-20a-5p	**1.02**	**4.11E-03**	0.23	4.07E-01	-0.05	9.47E-01	-	-
**hsa-miR-21-3p**	**1.05**	**9.65E-03**	0.70	**3.42E-02**	0.96	**2.16E-02**	**1.14**	**4.45E-02**
**hsa-miR-503-5p**	0.75	**1.26E-02**	**1.32**	**3.55E-06**	0.70	1.98E-01	0.26	1.59E-01
hsa-miR-17-5p	**1.02**	**1.36E-02**	0.17	4.93E-01	0.00	9.90E-01	-	-
hsa-miR-154-5p	**-1.10**	**2.01E-02**	0.61	**3.49E-02**	0.22	8.01E-01	-	-
hsa-miR-409-3p	**-1.33**	**2.19E-02**	0.17	6.23E-01	-0.13	9.18E-01	-	-
**hsa-miR-551b-3p**	**1.36**	**2.87E-02**	**1.52**	**3.84E-03**	**1.26**	3.16E-01	**1.98**	**6.75E-03**
hsa-miR-149-5p	**-1.32**	**3.50E-02**	-0.45	3.01E-01	-0.78	3.57E-01	-	-
**hsa-miR-96-5p**	**1.20**	**3.69E-02**	**3.51**	**6.51E-19**	**3.76**	**2.63E-15**	**2.26**	**1.74E-02**
hsa-miR-369-3p	**-1.00**	**4.23E-02**	0.45	1.85E-01	-0.16	9.15E-01	-	-
**hsa-miR-183-5p**	0.97	**4.39E-02**	**3.21**	**2.64E-13**	**4.06**	**2.00E-18**	**4.64**	**7.85E-03**
**hsa-miR-7-5p**	**-1.91**	**4.46E-02**	**-1.07**	**7.01E-04**	-0.46	5.20E-01	-0.13	1.42E-01
hsa-miR-663a	**1.02**	**4.46E-02**	NA	NA	NA	NA	-	-
**hsa-miR-542-3p**	0.46	8.46E-02	0.91	**2.34E-03**	**1.13**	**2.74E-02**	**-**	**-**
hsa-miR-31-5p	0.69	8.95E-02	**1.37**	**1.56E-03**	NA	NA	-	-
**hsa-miR-182-5p**	0.57	1.43E-01	**2.49**	**3.04E-09**	**3.59**	**1.99E-17**	**2.61**	**2.16E-02**
hsa-miR-337-3p	-0.72	1.54E-01	**1.05**	**1.02E-03**	0.36	6.34E-01	-	-
hsa-miR-509-3-5p	0.33	2.45E-01	**-2.80**	**1.13E-02**	**-2.68**	3.22E-01	-	-
hsa-miR-514a-3p	0.45	2.45E-01	**-2.79**	**1.42E-02**	**-2.10**	2.67E-01	-	-
hsa-miR-935	0.08	2.55E-01	NA	NA	**2.25**	**2.42E-03**	**-**	**-**
hsa-miR-199b-5p	0.60	3.16E-01	**1.34**	**4.44E-05**	0.72	3.49E-01	-	-
hsa-miR-129-5p	-0.58	3.29E-01	**-1.18**	**4.69E-02**	-0.39	8.33E-01	-	-
hsa-miR-383-5p	0.27	3.29E-01	NA	NA	**2.80**	**4.93E-02**	**-**	**-**
hsa-miR-301b	0.23	3.76E-01	**1.13**	**1.10E-05**	NA	NA	-	-
hsa-miR-767-5p	0.11	4.19E-01	-0.09	9.59E-01	**2.82**	**6.75E-04**	**-**	**-**
hsa-miR-489-3p	0.21	4.31E-01	**-4.13**	**9.94E-05**	NA	NA	-	-
hsa-miR-200c-3p	0.23	4.32E-01	0.21	3.94E-01	**6.39**	**2.09E-148**	**-**	**-**
hsa-miR-507	0.25	4.55E-01	**-2.36**	**3.71E-02**	NA	NA	-	-
hsa-miR-212-3p	0.37	4.86E-01	**-2.34**	**1.24E-05**	-0.72	5.20E-01	-	-
hsa-miR-132-3p	-0.46	4.88E-01	**-2.18**	**1.58E-05**	-0.75	4.88E-01	-	-
hsa-miR-653-5p	0.18	4.95E-01	**-3.73**	**2.28E-04**	**-1.94**	7.83E-02	-	-
hsa-miR-675-5p	0.17	4.95E-01	**-1.77**	**2.02E-02**	NA	NA	-	-
hsa-miR-132-5p	-0.21	5.46E-01	**-1.85**	**2.32E-04**	-0.75	4.88E-01	-	-
hsa-miR-508-3p	0.19	5.60E-01	**-2.62**	**1.72E-02**	**-1.64**	3.75E-01	-	-
hsa-miR-183-3p	0.21	6.55E-01	**2.63**	**7.95E-07**	NA	NA	-	-
**hsa-miR-202-5p**	0.19	6.66E-01	**-6.07**	**3.55E-06**	**-7.22**	**2.42E-03**	-1.40	**4.95E-03**
hsa-miR-450b-5p	0.20	6.78E-01	**1.23**	**1.59E-05**	0.90	8.56E-02	-	**-**
hsa-miR-217	-0.07	6.78E-01	-0.07	9.59E-01	**3.94**	**2.43E-26**	**-**	**-**
hsa-miR-204-5p	0.36	7.10E-01	**1.59**	**5.06E-05**	0.13	9.16E-01	-	-
hsa-miR-506-3p	0.17	7.42E-01	**-2.64**	**2.97E-02**	**-2.08**	4.88E-01	-	-
hsa-miR-196b-5p	0.23	7.51E-01	**1.51**	**1.14E-02**	0.03	9.47E-01	-	-
hsa-miR-216a-5p	-0.12	7.53E-01	0.07	8.74E-01	**3.81**	**1.31E-20**	**-**	**-**
hsa-miR-216b-5p	0.13	7.53E-01	-0.68	3.67E-01	**4.95**	**4.07E-21**	**-**	**-**
hsa-miR-122-5p	-0.13	7.91E-01	**-3.16**	**2.97E-03**	**-2.97**	3.20E-01	-	-
hsa-miR-1269a	NA	NA	NA	NA	**5.22**	**7.97E-13**	**-**	**-**
hsa-miR-653-3p	NA	NA	**-3.95**	**8.86E-05**	NA	NA	-	-
hsa-miR-513c-3p	NA	NA	**-2.85**	**1.61E-02**	NA	NA	-	-
hsa-miR-208a-3p	NA	NA	**-1.71**	**4.14E-02**	NA	NA	-	-
hsa-miR-3929	NA	NA	**1.74**	**8.86E-05**	NA	NA	-	-

miRNAs in bold are the miRNAs that fulfill the selection criteria. Cells in log_2 fold change (FC) columns are colored if $log_2FC > 0.75$ (in red) or < -0.75 (in green). False discovery rates (FDR) <0.05 are shown in red.

Table S4. Literature review of miRNAs that fulfill the selection criteria

miR studied	Literature references	Potential role according to literature	Status in PPGL *Discovery series*	Selected for validation
**miR-21-3p**	Potential oncomiR in renal, endometrial, ovarian, colorectal, oral, cervical, non-small cell lung cancers, and laryngeal carcinoma[1, 2, 11, 3–10]	Oncogene	Up	Yes
**miR-551b-3p**	Potential oncomiR in papillary thyroid carcinoma and ovarian cancer[12, 13]<br>Downregulated in gastric cancer[14]	Mostly oncogene	Up	Yes
**miR-202-5p**	Potential tumor suppressor in colorectal cancer, osteosarcoma[15–17]	Tumor suppressor	Down	Yes
**miR-7-5p**	Potential tumor suppressor in melanoma, glioblastoma, papillary thyroid and adrenocortical carcinoma, breast, bladder and prostate cancer[18, 19, 28, 20–27]<br>Upregulated in neuroendocrine neoplasms of the small intestine[29]	Mostly tumor suppressor	Down	Yes
**miR-542-3p**	Potential tumor suppressor in osteosarcoma, hepatocellular and oral squamous cell carcinomas, colorectal, non-small cell lung, esophageal, gastric and bladder cancers, neuroblastoma, melanoma, astrocytoma[30, 31, 40–47, 32–39]	Tumor suppressor	Up	No
**miR-503-5p**	Potential tumor suppressor in breast, colorectal, esophageal, gastric and non-small cell lung cancers, glioblastoma, glioma, hepatocellular carcinoma and osteosarcoma [48, 49, 58, 59, 50–57]<br>Potential oncomiR in colorectal and oesophageal cancers, glioblastoma[60–63]	?	Up	Yes
**miR-96-5p**<br>**miR-183-5p**<br>**miR-182-5p**	Already reported to be upregulated in PPGLs[64–67]	Oncogene	Up	Yes

Information obtained by quick reviewing abstracts from references obtained after performing the following advanced search of the PubMed database: "(*miRNA name*[Title/Abstract] AND cancer[Title/Abstract])". Last literature review: 28/August/2018.
Only miRNAs whose "potential role according to literature" was not in disagreement with "status in PPGL Discovery series" were selected for validation.

Table S5. Spearman's correlation (rho) between miR-21-3p and the expression of selected potential target genes in the Discovery and Validation series

miR-21-3p vs.	Discovery series								Validation series	
	Sub-series 1			Sub-series 2			Sub-series 3			
	rho	p-value	probe	rho	p-value	probe	rho	p-value	rho	p-value
CALM1	-0.32	2.33E-03	A_23_P163178	-0.42	2.00E-08	200653_s_at	-0.16	3.02E-02	0.06	6.71E-01
	-0.18	9.07E-02	A_24_P313186	-0.41	4.41E-08	213688_at				
CCND2	-0.09	4.07E-01	A_24_P270235	-0.45	1.37E-09	200952_s_at	-0.39	7.53E-08		
	-0.01	8.97E-01	A_23_P139881	-0.40	1.15E-07	200953_s_at				
CREBL2	-0.09	4.21E-01	A_24_P56194	-0.42	1.19E-08	201989_s_at	-0.24	1.51E-03	-0.07	6.26E-01
	-0.03	8.08E-01	A_23_P14026	-0.41	3.63E-08	201988_s_at				
KBTBD6	-0.07	5.21E-01	A_23_P392435	-0.40	7.12E-08	226479_at	-0.35	1.57E-06		
	-	-	-	-0.32	1.83E-05	1553111_a_at				
RICTOR	-0.07	4.98E-01	A_32_P193322	-0.32	2.24E-05	226310_at	-0.43	2.42E-09		
	0.10	3.59E-01	A_32_P15017	-0.26	5.55E-04	226312_at				
TSC2	0.02	8.45E-01	A_23_P66110	-0.40	5.94E-08	215735_s_at	-0.23	2.29E-03	-0.32	2.29E-02
	-	-	-	-0.21	6.05E-03	215624_at				
CETN3	-0.26	1.60E-02	A_23_P7732	-0.45	6.92E-10	209662_at	-0.35	1.85E-06		
	-	-	-	-	-	-				
KLHDC3	-0.29	6.74E-03	A_23_P156568	-0.43	7.80E-09	214383_x_at	-0.28	1.25E-04		
	-0.28	9.77E-03	A_24_P343255	-0.42	1.61E-08	208784_s_at				
PEX12	-0.44	2.10E-05	A_24_P416411	-0.23	3.05E-03	205094_at	-0.24	1.20E-03		
	-	-	-	-	-	-				
SGPL1	-0.27	1.23E-02	A_23_P75325	-0.28	1.88E-04	212321_at	-0.41	1.50E-08	-0.03	8.59E-01
	-0.18	9.69E-02	A_24_P940815	-0.28	1.96E-04	208381_s_at				

For each gene in sub-series 1 and 2, we only show the two probes, when available, with the most significant correlation.

Table S6. Spearman's correlation (rho) between miR-183-5p and the expression of selected potential target genes in the Discovery and Validation series.

miR-183-5p vs.	Discovery series								Validation series	
	Sub-series 1			Sub-series 2			Sub-series 3			
	rho	p-value	probe	rho	p-value	probe	rho	p-value	rho	p-value
RAI2	-0.41	1.08E-02	A_23_P254165	-0.36	2.26E-06	219440_at	-0.22	3.65E-03	NA	NA
	-	-	-	-	-	-				
SMAD7	-0.37	3.10E-03	A_23_P55518	-0.46	2.70E-10	204790_at	-0.25	6.99E-04	-0.17	2.3E-01
	-	-	-	-	-	-				
PLCB4	-0.19	3.65E-01	A_23_P28898	-0.45	9.34E-10	203895_at	-0.26	3.73E-04		
	-	-	-	-0.37	5.85E-07	203896_s_at				
STK35	-0.22	4.66E-02	A_23_P342600	-0.43	4.62E-09	225649_s_at	-0.14	6.08E-02		
	-0.14	3.60E-02	A_24_P940537	-0.40	1.13E-07	225648_at				

For each gene in sub-series 1 and 2, we only show the two probes, when available, with the most significant correlation.

Table S7. Literature review of potential miRNA target genes selected for validation

**Gene symbol, full gene name**[*]	**Protein-related function**[*]	**nº of ref. in PubMed**[Δ]	**Cancer-connected comments**[¤]	**Potential role according to literature**	**Selected for further study in PPGL**
***CALM1*, calmodulin 1**	Calcium-binding protein	16	Regulation of neuronal functions: neuronal differentiation, migration[68–70]	? but involved in neuronal roles	YES
***RAI2*, retinoic acid induced 2**	RAI2 function has not been characterized, but its expression is induced by retinoic acid that plays a critical role in development, cell growth, and differentiation	3	Tumor suppressor reported in breast and colorectal cancer[71–73]	Tumor suppressor	YES
***SMAD7*, SMAD family member 7**	Involved in regulation of the TGF-β pathway	310	Dual role in the regulation of cancer progression[74, 75], mostly reported to be a negative regulator of the pathway involved in epithelial-to-mesenchymal transition and metastasis[76]	Mostly tumor suppressor	YES
***PLCB4*, phospholipase C beta 4**	Catalyzes the formation of inositol trisphosphate and diacylglycerol from PIP2: involved in the transduction of signals in the retina	6	Gain-of function alterations reported in cancer[77–81]	Oncogene	NO
***STK35*, serine/threonine kinase 35**	Regulator of actin stress fibers in non-muscle cells	3	Might have an oncogenic role in osteosarcoma[82]	?	NO
***CETN3*, centrin 3**	Calcium binding-protein; it plays a fundamental role in centrosome duplication and separation	5	Loss of CETN3 connected to elevated EGFR[83]	?	NO
***KLHDC3*, kelch domain containing 3**	Involved in V(D)J and meiotic recombination, specifically expressed in testis	0	-	?	NO
***PEX12*, peroxisomal biogenesis factor 12**	Essential for the assembly of functional peroxisomes	1	-	?	NO
***SGPL1*, Sphingosine-1-Phosphate Lyase 1**	-	13	Sphingosine 1-phosphate (S1P) is a lipid with important roles in growth, survival and migration. It has been largely studied in cancer and SGPL1 is the phosphatase that irreversibly cleaves S1P and is the only exit point from sphingolipid pathways[84, 85]	Tumor suppressor	YES
***CCND2*, cyclin D2**	Forms a complex with CDK4/CDK6 and functions as a regulatory subunit of the complex, whose activity is required for cell cycle G1/S transition	398	Overexpression of cyclin D1 results in dysregulated CDK activity and cancer[86, 87]	Oncogene	NO
***CREBL2*, cAMP responsive element binding protein like 2**	DNA binding capabilities	2	Suggested as a tumor suppressor[88]	Likely tumor suppressor	YES
***KBTBD6*, kelch repeat and BTB domain containing 6**	-	0	-	?	NO
***RICTOR*, RPTOR independent companion of MTOR complex 2**	Protein complex that integrates nutrient- and growth factor-derived signals to regulate cell growth into mTOR pathway	200	Critical for mTORC2 activity and involved in tumorigenesis and metastasis[89]	Oncogene	NO
***TSC2*, TSC complex subunit 2**	Tumor suppressor gene involved in mTOR signaling	357	Together with TSC1 inhibits mTORC1 signalling[89, 90]	Tumor suppressor	YES

Last literature review: 30/August/2018

[*] Retrieved from Gene database from NCBI (https://www.ncbi.nlm.nih.gov/gene/)

[Δ] Number of reference results that appeared after performing the following advanced search of the PubMed database: "(*gene symbol*[Title/Abstract]) OR ("*full gene name*"[Title/Abstract]) AND cancer[Title/Abstract]". By doing this search we aimed to identify those genes that have been largely reported to be involved in cancer.

[¤] Information obtained by quick reviewing abstracts from references obtained in [Δ], which might be useful to establish a potential role of the gene in cancer.

Only those miRs whose "potential role according to literature" was in agreement with a potential involvement in the disease were selected for further study.

Table S8. List of mPPGL patients included in the exploratory analysis of circulating miRNAs

Sample ID	Mutation	Clinical data	Clinical status at the moment of sample collection	Metastatic burden (cm^3)
0014	SDHB	Bone metastases with spinal compression and elevated catecholamines. Clinical progression after treatment with evidence of new lesions. MIBG therapy, sunitinib, temozolamide, sandostatin, radiotherapy. Three bone metastases in July 2009. Last follow-up August 2016.	Progressive	12.192
0031	Sporadic	Mediastinal PGL and two interaortocaval PGLs (2 cm). Sample collected before surgery. No evidence of progression afterwards. Last follow-up in 2013)	Stable	4.578
0046	Sporadic	Diagnosis of PCC in 2008; relapse and nephrectomy in 2010. Sunitinib treatment 2012-2015. Multiple nodal and peritoneal metastases in 2016.	Progressive	17.1685
0103	Sporadic	Pulmonary lesion in 2005 treated with surgery and chemotherapy. Diagnosis of PGL (3.7 cm) in 2008. No evidence of metastases.	Stable	0
0256	Sporadic	Diagnosis of metastatic PCC (bone). No evidence of progression at the moment of sample collection.	Stable	30
0309	Sporadic	Diagnosis of H&N PGL in 1990, treated with surgery and radiotherapy. Relapse with bone, liver and pulmonary lesions in 2001, treated with chemotherapy and radiotherapy. Cementoplasty of bone metastases in July 2014.	Progressive	434.487
0345	Sporadic	Diagnosis of PCC (9 cm); no metastases detected before surgery. Pulmonary metastases appeared afterwards.	Stable	0
0566	SDHB	Diagnosis of metastatic PGL in 2003 (node, bone, liver and pulmonary lesions). Treatment with surgery and chemotherapy. Ablation of liver lesions in 2006. Sunitinib treatment 2015-2016 with decrease of pulmonary and node lesions according to FDG PET scan. Clinically stable by 2017.	Stable	87.789
0638	SDHA	Diagnosis of PGL in 2010 treated with surgery. Diaphragmatic lesions appeared in 2013 treated with surgery and chemotherapy. Three abdominal masses appeared in 2015. Last follow-up in 2017.	Progressive	19.6105
0653	SDHB	Metastatic PGL (bone and nodes). Laminectomy in march 2016; no progression afterwards.	Stable	N.A.

Two subgroups of mPPGL patients (progressive or stable) were defined according to the clinical condition of the patient at the moment of blood draw as judged by the treating physician. Metastatic burden (total volume of the metastatic lesions) was determined on the basis of radiological evaluation by two experts (C.L.L and A.B.). The corresponding genotype and relevant clinical information for each patient with regard to the treatment received are also indicated. N.A: not analyzed.

1. Doberstein K, Bretz NP, Schirmer U et al. MiR-21-3p is a positive regulator of L1CAM in several human carcinomas. Cancer Lett. 2014; 354(2):455-66.

2. Wang X, Chen L, Jin H et al. Screening miRNAs for early diagnosis of colorectal cancer by small RNA deep sequencing and evaluation in a chinese patient population. Onco. Targets. Ther. 2016; 9:1159-66.

3. Cybula M, Wieteska Łukasz, Józefowicz-Korczyńska M et al. New miRNA expression abnormalities in laryngeal squamous cell carcinoma. Cancer Biomarkers 2016; 16(4):559-68.

4. Jiang M, Zhang P, Hu G et al. Relative expressions of miR-205-5p, miR-205-3p, and miR-21 in tissues and serum of non-small cell lung cancer patients. Mol. Cell. Biochem. 2013; 383(1-2):67-75.

5. Lu ZM, Lin YF, Jiang L et al. Micro-ribonucleic acid expression profiling and bioinformatic target gene analyses in laryngeal carcinoma. Onco. Targets. Ther. 2014; 7:525-33.

6. Han Y, Xu G-X, Lu H et al. Dysregulation of miRNA-21 and their potential as biomarkers for the diagnosis of cervical cancer. Int. J. Clin. Exp. Pathol. 2015; 8(6):7131-9.

7. Zeljic K, Jovanovic I, Jovanovic J et al. MicroRNA meta-signature of oral cancer: evidence from a meta-analysis. Ups. J. Med. Sci. 2018; 123(1):43-49.

8. Hou N, Guo Z, Zhao G et al. Inhibition of microRNA-21-3p suppresses proliferation as well as invasion and induces apoptosis by targeting RBPMS through Smad4/ERK signaling pathway in human colorectal cancer HCT116 cells. Clin. Exp. Pharmacol. Physiol. 2018; 45(7):729-741.

9. Jiao W, Leng X, Zhou Q et al. Different miR-21-3p isoforms and their different features in colorectal cancer. Int. J. Cancer 2017; 141(10):2103-2111.

10. Veterans K, Hospital G, Tzu T et al. Next-generation Sequencing for microRNA Profiling: MicroRNA-21-3p Promotes Oral Cancer Metastasis. Anticancer Res. 2017; 37(3):1059–1066.

11. Báez-Vega PM, Echevarría Vargas IM, Valiyeva F et al. Targeting miR-21-3p inhibits proliferation and invasion of ovarian cancer cells. Oncotarget 2016; 7(24):36321-36337.

12. Swierniak M, Wojcicka A, Czetwertynska M et al. In-depth characterization of the MicroRNA transcriptome in normal thyroid and papillary thyroid carcinoma. J. Clin. Endocrinol. Metab. 2013; 98(8):E1401-9.

13. Chaluvally-Raghavan P, Jeong KJ, Pradeep S et al. Direct Upregulation of STAT3 by MicroRNA-551b-3p Deregulates Growth and Metastasis of Ovarian Cancer. Cell Rep. 2016; 15(7):1493-1504.

14. Chen Z, Liu X, Hu Z et al. Identification and characterization of tumor suppressor and oncogenic miRNAs in gastric cancer. Oncol. Lett. 2015; 10(1):329-336.

15. Ke S-B, Qiu H, Chen J-M et al. MicroRNA-202-5p functions as a tumor suppressor in colorectal carcinoma by directly targeting SMARCC1. Gene 2018; 676:329-335.

16. Li C, Ma D, Yang J et al. miR-202-5p inhibits the migration and invasion of osteosarcoma cells by targeting ROCK1. Oncol. Lett. 2018:829–834.

17. zhang J, Li X-Y, Hu P, Ding Y-S. LncRNA NORAD contributes to colorectal cancer progression by inhibition of miR-202-5p. Oncol. Res. Featur. Preclin. Clin. Cancer Ther. 2018; (17):1–18.

18. Giles KM, Brown RAM, Epis MR et al. MiRNA-7-5p inhibits melanoma cell migration and invasion. Biochem. Biophys. Res. Commun. 2013; 430(2):706-10.

19. Dong L, Li Y, Han C et al. miRNA microarray reveals specific expression in the peripheral blood of glioblastoma patients. Int. J. Oncol. 2014; 45(2):746-56.

20. Liu Z, Liu Y, Li L et al. MiR-7-5p is frequently downregulated in glioblastoma microvasculature and inhibits vascular endothelial cell proliferation by targeting RAF1. Tumor Biol. 2014; 35(10):10177-84.

21. Saiselet M, Gacquer D, Spinette A et al. New global analysis of the microRNA transcriptome of primary tumors and lymph node metastases of papillary thyroid cancer. BMC Genomics 2015; 16:828.

22. Shi Y, Luo X, Li P et al. MiR-7-5p suppresses cell proliferation and induces apoptosis of breast cancer cells mainly by targeting

REGγ. Cancer Lett. 2015; 358(1):27-36.

23. Li J, Qiu M, An Y et al. miR-7-5p acts as a tumor suppressor in bladder cancer by regulating the hedgehog pathway factor Gli3. Biochem. Biophys. Res. Commun. 2018:1–7.

24. Zhu W, Wang Y, Zhang D et al. MiR-7-5p functions as a tumor suppressor by targeting SOX18 in pancreatic ductal adenocarcinoma. Biochem. Biophys. Res. Commun. 2018; 497(4):963–970.

25. Jahanbani I, Al-Abdallah A, Ali R et al. Discriminatory miRNAs for the management of papillary thyroid carcinoma and noninvasive follicular thyroid neoplasms with papillary-like nuclear features. Thyroid 2018; 28(3):319-327.

26. Zhang L, Qi M, Feng T et al. IDH1R132H Promotes Malignant Transformation of Benign Prostatic Epithelium by Dysregulating MicroRNAs: Involvement of IGF1R-AKT/STAT3 Signaling Pathway. Neoplasia 2018; 20(2):207-217.

27. Giles KM, Brown RAM, Ganda C et al. microRNA-7-5p inhibits melanoma cell metastasis by suppressing RelA / NF-κB proliferation and metastasis by suppressing RelA/NF-KB. 2016; 7(22):31663-80.

28. Glover AR, Zhao JT, Gill AJ et al. microRNA-7 as a tumor suppressor and novel therapeutic for adrenocortical carcinoma. Oncotarget 2015; 6(34):36675-88.

29. Heverhagen AE, Legrand N, Wagner V et al. Overexpression of MicroRNA miR-7-5p Is a Potential Biomarker in Neuroendocrine Neoplasms of the Small Intestine. Neuroendocrinology 2018; 106(4):312–317.

30. Wu Y, You J, Li F et al. MicroRNA-542-3p suppresses tumor cell proliferation via targeting Smad2 inhuman osteosarcoma. Oncol. Lett. 2018:6895–6902.

31. Zhang T, Liu W, Meng W et al. Downregulation of miR-542-3p promotes cancer metastasis through activating TGF-beta/Smad signaling in hepatocellular carcinoma. Onco. Targets. Ther. 2018; 11:1929–1939.

32. Yang C, Wang M-H, Zhou J-D, Chi Q. Upregulation of miR-542-3p inhibits the growth and invasion of human colon cancer cells through PI3K/AKT/survivin signaling. Oncol. Rep. 2017; 38(6):3545-3553.

33. Qiao B, Cai J-H, King-Yin Lam A, He B-X. MicroRNA-542-3p inhibits oral squamous cell carcinoma progression by inhibiting ILK/TGF-β1/Smad2/3 signaling. Oncotarget 2017; 8(41):70761–70776.

34. Liu B, Li J, Zheng M et al. MiR-542-3p exerts tumor suppressive functions in non-small cell lung cancer cells by upregulating FTSJ2. Life Sci. 2017; 188:87–95.

35. Tao J, Liu Z, Wang Y et al. MiR-542-3p inhibits metastasis and epithelial-mesenchymal transition of hepatocellular carcinoma by targeting UBE3C. Biomed. Pharmacother. 2017; 93:420–428.

36. Yuan L, Yuan P, Yuan H et al. miR-542-3p inhibits colorectal cancer cell proliferation, migration and invasion by targeting OTUB1. Am. J. Cancer Res. 2017; 7(1):159–172.

37. Wu H-X, Wang G-M, Lu X, Zhang L. miR-542-3p targets sphingosine-1-phosphate receptor 1 and regulates cell proliferation and invasion of breast cancer cells. Eur. Rev. Med. Pharmacol. Sci. 2017; 21(1):108–114.

38. He T, Qi F, Jia L et al. MicroRNA-542-3p inhibits tumour angiogenesis by targeting Angiopoietin-2. J. Pathol. 2014; 232(5):499-508.

39. Althoff K, Lindner S, Odersky A et al. miR-542-3p exerts tumor suppressive functions in neuroblastoma by downregulating survivin. Int. J. Cancer 2015; 136(6):1308-20.

40. Zhang J, Wang S, Han F et al. MicroRNA-542-3p suppresses cellular proliferation of bladder cancer cells through post-transcriptionally regulating survivin. Gene 2016; 579(2):146-22.

41. Wu W, Dang S, Feng Q et al. MicroRNA-542-3p inhibits the growth of hepatocellular carcinoma cells by targeting FZD7/Wnt signaling pathway. Biochem. Biophys. Res. Commun. 2017; 482(1):100–105.

42. Rang Z, Yang G, Wang YW, Cui F. MIR-542-3p suppresses invasion and metastasis by targeting the proto-oncogene serine/threonine protein kinase, PIM1, in melanoma. Biochem. Biophys. Res. Commun. 2016; 474(2):315-320.

43. Long HC, Gao X, Lei CJ et al. MiR-542-3p inhibits the growth and invasion of colorectal cancer cells through targeted regulation of cortactin. Int. J. Mol. Med. 2016; 37(4):1112–1118.

44. Cai J, Zhao J, Zhang N et al. MicroRNA-542-3p Suppresses Tumor Cell Invasion via Targeting AKT Pathway in Human Astrocytoma. J. Biol. Chem. 2015; 290(41):24678-88.

45. Shen X, Si Y, Yang Z et al. MicroRNA-542-3p suppresses cell growth of gastric cancer cells via targeting oncogene astrocyte-elevated gene-1. Med. Oncol. 2015; 32(1):1–8.

46. Oneyama C, Morii E, Okuzaki D et al. MicroRNA-mediated upregulation of integrin-linked kinase promotes Src-induced tumor progression. Oncogene 2012; 31(13):1623-35.

47. Yoon S, Choi YC, Lee S et al. Induction of growth arrest by miR-542-3p that targets survivin. FEBS Lett. 2010; 584(18):4048-52.

48. Long J, Ou C, Xia H et al. MiR-503 inhibited cell proliferation of human breast cancer cells by suppressing CCND1 expression. Tumor Biol. 2015; 36(11):8697–8702.

49. Chang S-W, Yue J, Wang B-C, Zhang X-L. miR-503 inhibits cell proliferation and induces apoptosis in colorectal cancer cells by targeting E2F3. Int. J. Clin. Exp. Pathol. 2015; 8(10):12853-60.

50. Jiang L, Zhao Z, Zheng L et al. Downregulation of miR-503 Promotes ESCC Cell Proliferation, Migration, and Invasion by Targeting Cyclin D1. Genomics, Proteomics Bioinforma. 2017; 15(3):208–217.

51. PENG Y, LIU Y-M, LI L-C et al. microRNA-503 inhibits gastric cancer cell growth and epithelial-to-mesenchymal transition. Oncol. Lett. 2014; 7(4):1233–1238.

52. Zhang Y, Chen X, Lian H et al. MicroRNA-503 acts as a tumor suppressor in glioblastoma for multiple antitumor effects by targeting IGF-1R. Oncol. Rep. 2014; 31(3):1445-52.

53. Liu H, Song Z, Liao D et al. miR-503 inhibits cell proliferation and invasion in glioma by targeting L1CAM. Int. J. Clin. Exp. Med. 2015; 8(10):18441-7.

54. Xiao F, zhang W, Chen L et al. MicroRNA-503 inhibits the G1/S transition by downregulating cyclin D3 and E2F3 in hepatocellular carcinoma. J. Transl. Med. 2013; 11:195.

55. Li B, Liu L, Li X, Wu L. MiR-503 suppresses metastasis of hepatocellular carcinoma cell by targeting PRMT1. Biochem. Biophys. Res. Commun. 2015; 464(4):982-987.

56. Liu L, Qu W, Zhong Z. Down-regulation of miR-503 expression predicate advanced mythological features and poor prognosis in patients with NSCLC. Int. J. Clin. Exp. Pathol. 2015; 8(5):5609-13.

57. Chong Y, Zhang J, Guo X et al. MicroRNA-503 Acts as a tumor suppressor in osteosarcoma by targeting L1CAM. PLoS One 2014; 9(12):e114585.

58. Guo X, Zhang J, Pang J et al. MicroRNA-503 represses epithelial–mesenchymal transition and inhibits metastasis of osteosarcoma by targeting c-myb. Tumor Biol. 2016; 37(7):9181–9187.

59. Guo J, Liu X, Wang M. MiR-503 suppresses tumor cell proliferation and metastasis by directly targeting RNF31 in prostate cancer. Biochem. Biophys. Res. Commun. 2015; 464(4):1302-1308.

60. Oneyama C, Kito Y, Asai R et al. MiR-424/503-Mediated rictor upregulation promotes tumor progression. PLoS One 2013; 8(11):e80300.

61. Noguchi T, Toiyama Y, Kitajima T et al. miRNA-503 Promotes Tumor Progression and Is Associated with Early Recurrence and Poor Prognosis in Human Colorectal Cancer. Oncology 2016; 90(4):221-31.

62. Ide S, Toiyama Y, Shimura T et al. MicroRNA-503 promotes tumor progression and acts as a novel biomarker for prognosis in oesophageal cancer. Anticancer Res. 2015; 35(3):1447-51.

63. Guo P, Yu Y, Li H et al. TGF-â1-induced miR-503 controls cell growth and apoptosis by targeting PDCD4 in glioblastoma cells. Sci. Rep. 2017; 7(1):1–10.

64. Castro-Vega LJ, Letouze E, Burnichon N et al. Multi-omics analysis defines core genomic alterations in pheochromocytomas and paragangliomas. Nat Commun 2015; 6:6044.

65. De Cubas AA, Leandro-Garc??a LJ, Schiavi F et al. Integrative analysis of miRNA and mRNA expression profiles in pheochromocytoma and paraganglioma identifies genotype-specific markers and potentially regulated pathways. Endocr.

Relat. Cancer 2013; 20(4):477–493.

66. Pillai S, Lo CY, Liew V et al. MicroRNA 183 family profiles in pheochromocytomas are related to clinical parameters and SDHB expression. Hum. Pathol. 2017; 64:91–97.

67. Patterson E, Webb R, Weisbrod A et al. The microRNA expression changes associated with malignancy and SDHB mutation in pheochromocytoma. Endocr. Relat. Cancer 2012; 19(2):157–166.

68. Wang B, Pan L, Wei M et al. FMRP-Mediated Axonal Delivery of miR-181d Regulates Axon Elongation by Locally Targeting Map1b and Calm1. Cell Rep. 2015; 13(12):2794-807.

69. Kobayashi H, Saragai S, Naito A et al. Calm1 signaling pathway is essential for the migration of mouse precerebellar neurons. Development 2015; 142(2):375-84.

70. Toutenhoofd SL, Strehler EE. Regulation of calmodulin mRNAs in differentiating human IMR-32 neuroblastoma cells. Biochim. Biophys. Acta. 2002; 1600(1-2):95-104.

71. Esposito M, Kang Y. RAI2: Linking retinoic acid signaling with metastasis suppression. Cancer Discov. 2015; 5(5):466-8.

72. Yan W, Wu K, Herman JG et al. Retinoic acid-induced 2 (RAI2) is a novel tumor suppressor, and promoter region methylation of RAI2 is a poor prognostic marker in colorectal cancer. Clin. Epigenetics 2018; 10:69.

73. Werner S, Brors B, Eick J et al. Suppression of early hematogenous dissemination of human breast cancer cells to bone marrow by retinoic acid–induced 2. Cancer Discov. 2015; 5(5):506-19.

74. Stolfi C, Marafini I, De Simone V et al. The dual role of Smad7 in the control of cancer growth and metastasis. Int. J. Mol. Sci. 2013; 14(12):23774-90.

75. Luo L, Li N, Lv N, Huang D. SMAD7: a timer of tumor progression targeting TGF-β signaling. Tumor Biol. 2014; 35(9)8379-85.

76. Massagué J. TGFβ signalling in context. Nat. Rev. Mol. Cell Biol. 2012; 13(10):616-30.

77. Waugh MG. Chromosomal Instability and Phosphoinositide Pathway Gene Signatures in Glioblastoma Multiforme. Mol. Neurobiol. 2014; 53(1):621-630.

78. Ma D, Yang J, Wang Y et al. Whole exome sequencing identified genetic variations in Chinese hemangioblastoma patients. Am. J. Med. Genet. Part A 2017; 173(10):2605-2613.

79. van de Nes JAP, Koelsche C, Gessi M et al. Activating CYSLTR2 and PLCB4 Mutations in Primary Leptomeningeal Melanocytic Tumors. J. Invest. Dermatol. 2017; 137(9):2033-2035.

80. Li C-F, Liu T-T, Chuang I-C et al. PLCB4 copy gain and PLCß4 overexpression in primary gastrointestinal stromal tumors: Integrative characterization of a lipid-catabolizing enzyme associated with worse disease- free survival. Oncotarget 2017; 8(12):19997-20010.

81. Johansson P, Aoude LG, Wadt K et al. Deep sequencing of uveal melanoma identifies a recurrent mutation in PLCB4. Oncotarget 2016; 7(4):4624-31.

82. Wu Z, Liu J, Hu S et al. Serine/threonine kinase 35, a target gene of stat3, regulates the proliferation and apoptosis of osteosarcoma cells. Cell. Physiol. Biochem. 2018; 45(2):808–818.

83. Mertins P, Mani DR, Ruggles K V. et al. Proteogenomics connects somatic mutations to signalling in breast cancer. Nature 2016; 534(7605):55-62.

84. Spiegel S, Milstien S. Sphingosine-1-phosphate: An enigmatic signalling lipid. Nat. Rev. Mol. Cell Biol. 2003; 4(5):397-407.

85. Pyne NJ, El Buri A, Adams DR, Pyne S. Sphingosine 1-phosphate and cancer. Adv. Biol. Regul. 2018; 68(7):97–106.

86. Qie S, Diehl JA. Cyclin D1, cancer progression, and opportunities in cancer treatment. J. Mol. Med. 2016; 94(12):1313-1326.

87. Malumbres M, Barbacid M. Cell cycle, CDKs and cancer: A changing paradigm. Nat. Rev. Cancer 2009; 9(3):153-66.

88. Hoornaert I, Marynen P, Baens M. CREBL2, a novel transcript from the chromosome 12 region flanked by ETV6 and CDKN1B. Genomics 1998; 51(1):154-7.

89. Zoncu R, Efeyan A, Sabatini DM. mTOR: from growth signal integration to cancer, diabetes and ageing. Nat. Rev. Mol. Cell

Biol. 2011; 12(1):21-35.

90. Inoki K, Li Y, Zhu T et al. TSC2 is phosphorylated and inhibited by Akt and suppresses mTOR signalling. Nat. Cell Biol. 2002; 4(9):648-57.

SUPPLEMENTARY METHODS

Data availability

Sub-series	miRNA profiling platform	Nº of samples	Repository	Reference
1	Agilent-019118 Human miRNA Microarray 2.0 G4470B	93	GEO (GSE29742)	De Cubas *et al.* [1]
2	RNA-seq (Illumina HiSeq2000 sequencer)	171	ArrayExpress (E-MTAB-2833)	Castro-Vega *et al.* [2]
3	RNA-seq (Illumina HiSeq2000 sequencer)	179	TCGA Data Portal (https://portal.gdc.cancer.gov/)	Fishbein *et al.* [3]
	**mRNA profiling platform**			
1	Agilent-014850 Whole Human Genome Microarray 4x44K G4112F	109	GEO (GSE19422, GSE51081)	López-Jiménez *et al.* [4]; Qin *et al.* [5]
2	Affymetrix (GeneChip Human Genome U133 Plus 2.0)	177	ArrayExpress (E-MTAB-733)	Castro-Vega *et al.* [2]
3	RNA-seq (Illumina HiSeq2000 sequencer)	179	TCGA Data Portal (https://portal.gdc.cancer.gov/)	Fishbein *et al.* [3]

Discovery of differentially expressed miRNAs

Merging the three datasets was not possible since different platforms had been used, and therefore they were treated as three sub-series of a larger discovery series as specified bellow:

Sub-series 1: Microarray image acquisition and analysis was done using a G2505C microarray scanner (Agilent). The text files with the data of the processed images were analyzed with *limma* R package (version 3.26.9). Background correction was done using the "normexp" method. Normalization between arrays was done applying the "quantile" method. Values for within-array replicate probes were replaced with their average by using the *avereps* function of limma.

After estimating the fold changes and standard errors by fitting a linear model for each probe and applying the empirical Bayes to smooth the standard errors, a list of probes was obtained that were differentially expressed between metastatic and non-metastatic samples. The miRNA names corresponding to the probes were converted to those of release 20 of miRBase [6] by using an in-house Perl script that processed the conversion files downloaded from miRBase Tracker [7].

Sub-series 2: The miRNA read files of the samples in FastQ format were aligned to release 20 of miRBase [6] using *bowtie* [8] (version 0.12.7) with seed length equal to 17; no mismatches were allowed in the seed and at most one alignment per read was allowed. *Htseq-count* [9] (version 0.5.3p9) was used to generate the read count data for each miRNA.

Only miRNAs having a minimum of 15 counts in at least 7.3 % of the samples in both the subset of metastatic samples and the subset of non-metastatic samples were selected for further the analysis.

The *edgeR* package (version 3.12.1) [10] was then used to normalize the matrix using the trimmed mean of M-values (TMM) [11] and extract the list of differentially expressed genes by using the *exactTest* function.

Only miRNAs with an average count >100 in any of the groups were considered further.

Sub-series 3: An in-house Perl script was used to a) sum all the read counts corresponding to all isoforms of each mature miRNA accession number; b) convert the mature miRNA sequence names into the names in release 20 of miRBase; and c) generate a matrix containing the counts of all miRNAs in all input TCGA samples.

Only miRNAs having a minimum of 15 counts in at least 5.4 % of the samples in both subsets of metastatic and non-metastatic samples were selected for further analysis.

The R package *edgeR* was then used to normalize the matrix using the TMM and obtain the genes differentially expressed between non-metastatic and metastatic samples by using the *exactTest* function.

Only miRNAs with an average count >100 in any of the groups were considered further, as they were deemed easily detectable markers.

Filtering criteria for selecting miRNAs for validation: A list of significantly differentially expressed miRNAs was obtained by considering only those miRNAs with an FDR<0.05 and a $|\log_2$ fold change$| \geq 0.75$. Only miRNAs shared by at least two sub-series were selected for further consideration. In addition to the above criteria, we performed an exhaustive literature review, which included two mining resources (http://mircancer.ecu.edu/index.jsp and http://lifeome.net/database/oncomirdb/) as well as a search of PubMed abstracts (https://www.ncbi.nlm.nih.gov/pubmed/) using keywords. A relation between the miRNAs and cancer reported in the literature was required for the final selection of candidates for validation.

Confirmation of differentially expressed miRNAs in a validation series

RNA was extracted from formalin-fixed paraffin-embedded (FFPE) sections of the tumor tissue samples using the RNeasy FFPE kit (Qiagen, Hilden, Germany) following the manufacturer's instructions. cDNA was synthesized by reverse transcription (RT) of total RNA using the miRCURY LNA™ Universal RT miR PCR kit (Exiqon, Vedbaek, Denmark) according to manufacturer's instructions. The resulting cDNA was diluted 1:40 and PCR reactions were carried out using the ExiLENT SYBR® Green Master Mix kit (Exiqon, Vedbaek, Denmark) and microRNA LNA™ PCR primer sets (Exiqon, Vedbaek, Denmark). Stably expressed endogenous control 5S rRNA (CV of all samples=11%) was used for data normalization. The limit of detection of the instrument (Ct=40) was assigned to the samples with no detectable expression.

All qPCR reactions were performed on a QuantStudio™ 6-7 Flex Real-Time PCR System (Applied Biosystems, Foster City, California). Blanks and controls were included in all PCR series and reactions were carried out in triplicate. Expression of each miRNA was calculated with the ΔΔCt method using the Ct value of the endogenous control to normalize the data[12].

Integration of miRNA and mRNA expression profiles of the discovery series

Step 2. Generation of normalized mRNA expression matrices including only step 1 genes (Targetome) for each sub-series was performed as specified below.

Sub-series 1: Microarray image acquisition and analysis was done with GenePix™ Pro (Axon Instruments Inc). The produced .gpr files were read and analyzed with *limma* R package [13] (version 3.26.9). Background correction was done using the "normexp" method. Normalization within arrays was performed using the "loess" method. Normalization between arrays was done applying the "quantile" method.

Sub-series 2: We downloaded the Affymetrix (GeneChip Human Genome U133 Plus 2.0) intensity (.CEL) files of 177 samples from ArrayExpress (https://www.ebi.ac.uk/arrayexpress/) experiment ID E-MTAB-733. We read and processed these .CEL files using the *affy* and *limma* packages of R (versions 1.48.0 and 3.26.9, respectively). Background correction was done using the "rma" method, and the normalization method used was "quantiles".

Sub-series 3: An in-house Perl script collapsed these files into a unique matrix containing the raw counts for all samples. R package *edgeR* (version 3.12.1) was then used to normalize the matrix using the TMM.

Generation of targetome matrices: For each series, miRNA and mRNA expression submatrices were obtained from the miRNA and mRNA expression matrices previously generated. These submatrices contained the expression values for the sample codes shared by the miRNA expression matrix and the mRNA expression matrix. The number of sample codes shared by miRNA expression and mRNA expression matrices in each series was 87 for sub-series 1, 168 for sub-series 2, and 179 for sub-series 3.

Next, a third submatrix was generated from these submatrices for each series, containing only those genes that were included in the lists generated in Step 1 (potential gene targets for either miR-21-3p or miR-183-5p), which we designated *targetome* of miR-21-3p and *targetome* of miR-183-5p.

qPCR

Regarding the cell model, 100,000 cells of each cell line were seeded in duplicate in 6 well-plates. One of the duplicates was seeded using complete medium, and the other using complete medium plus 1µg/ml doxycycline (Sigma#D9891). RNA was extracted using TRI Reagent® (MRC#TR 118) following the vendor's instructions at different time points (0, 24, 48, 84, 144 and 168 h after plating). Medium was changed after 84h. In the validation series we used the RNA that had been extracted for miRNA quantification and validation in this series.

In all cases, cDNAs were prepared from 500ng of RNA using the qScript™ cDNA Synthesis Kit (#95047-100, Quanta Biosciences, Gaithersburg, MD) and mRNA levels were quantified by real-time PCR using the

Universal ProbeLibrary set (Roche), as described by the vendor, on a QuantStudio 6 Flex Real-Time PCR System (Applied Biosystems). Normalization was carried out with the β-actin housekeeping gene and relative mRNA levels were estimated by the ΔΔCt method [12].

Detection of circulating miRNA

Droplet Digital PCR. Two µl of input RNA were used for universal reverse transcription reactions (TaqMan® Advanced miRNA cDNA Synthesis Kit, Thermo Fisher Scientific), and pre-amplified following the manufacturer's recommendations. For ddPCR, a reaction mix of 5 µl cDNAs (diluted 1:10 or 1:50), 10 µl ddPCR master mix (2X), 1 µl specific TaqMan® Advanced miRNA Assays (20X) and 4 µl RNase-free water was mixed with 70 µl of QX100 Droplet Generation oil and placed into the QX100™ Droplet Generator (Bio-Rad). In total, 40 µl of droplets were transferred to a 96-well PCR plate and PCR amplifications were carried out in a Bio-Rad C1000 thermal cycler using the following cycling conditions: enzyme activation at 95°C, 10 min; 40 cycles of denaturation at 95°C for 3 sec, annealing / extension at 60°C for 30 sec, and a final step of inactivation at 98°C for 10 min. After PCR, 96-well plates were loaded onto a QX200 Droplet Reader, which counts the positive droplets containing amplification products and negative droplets without amplification products, to calculate target concentration (copies/µl PCR reaction) using Poisson statistics. Fluorescence amplitude thresholds were determined manually using the 2D amplitude chart. All TaqMan® assays were tested for linearity in serial dilutions (1:4) of RT products generated from a pool of RNAs. Spike-in cel-miR-39-3p (Thermo Fisher Scientific) was added as internal reference to adjust for differences of miRNA recovery between samples.

1. De Cubas AA, Leandro-García LJ, Schiavi F et al. Integrative analysis of miRNA and mRNA expression profiles in pheochromocytoma and paraganglioma identifies genotype-specific markers and potentially regulated pathways. Endocr. Relat. Cancer 2013; 20(4):477–493.

2. Castro-Vega LJ, Letouze E, Burnichon N et al. Multi-omics analysis defines core genomic alterations in pheochromocytomas and paragangliomas. Nat Commun 2015; 6:6044.

3. Fishbein L, Leshchiner I, Walter V et al. Comprehensive Molecular Characterization of Pheochromocytoma and Paraganglioma. Cancer Cell 2017; 31(2):181–193.

4. López-Jiménez E, Gómez-López G, Leandro-García LJ et al. Research Resource: Transcriptional Profiling Reveals Different Pseudohypoxic Signatures in SDHB and VHL-Related Pheochromocytomas. Mol. Endocrinol 2010; 24(12):2382-91.

5. Qin N, De Cubas AA, Garcia-Martin R et al. Opposing effects of HIF1α and HIF2α on chromaffin cell phenotypic features and tumor cell proliferation: Insights from MYC-associated factor X. Int. J. Cancer 2014; 135(9):2054-64.

6. Kozomara A, Griffiths-Jones S. MiRBase: Annotating high confidence microRNAs using deep sequencing data. Nucleic Acids Res 2014; 42:D68-73.

7. Van Peer G, Lefever S, Anckaert J et al. miRBase Tracker: keeping track of microRNA annotation changes. Database (Oxford). 2014. doi:10.1093/database/bau080.

8. Langmead B, Trapnell C, Pop M, Salzberg S. Ultrafast and memory-efficient alignment of short DNA sequences to the human genome. Genome Biol. 2009; 10(3):R25.

9. Anders S, Pyl PT, Huber W. HTSeq-A Python framework to work with high-throughput sequencing data. Bioinformatics 2015; 31(2):166-9.

10. Robinson MD, Oshlack A. A scaling normalization method for differential expression analysis of RNA-seq data. Genome Biol. 2010; 11(3):R25

11. Robinson MD, McCarthy DJ, Smyth GK. edgeR: a Bioconductor package for differential expression analysis of digital gene expression data. Bioinformatics 2010; 26(1):139-40.

12. Livak KJ, Schmittgen TD. Analysis of relative gene expression data using real-time quantitative PCR and the 2(-Delta Delta C(T)) Method. Methods 2001; 25(4):402–8.

13. Ritchie ME, Phipson B, Wu D et al. Limma powers differential expression analyses for RNA-sequencing and microarray studies. Nucleic Acids Res. 2015; 43(7):e47.
